# Supplementary material for: Estimating the global health impact of gender-based violence and violence against children: a systematic review and meta-analysis protocol
Source: BMJ Open. 2022 Jun 27;12(6):e061248. doi: 10.1136/bmjopen-2022-061248 (PMC9240882; doi:10.1136/bmjopen-2022-061248)
Supplement: Supplementary data [file bmjopen-2022-061248supp002.pdf]

Supplementary Table 1. Search Terms for Embase.

	Concept: Violence	Concept: Study type	Concept: Risk
Subject Headings (Emtree)	'sexual violence'/exp<br>'forced sex'/exp<br>'violence'/de<br>'domestic violence'/exp<br>'gender based violence'/exp<br>'partner violence'/exp<br>'dating violence'/exp<br>'physical abuse'/exp<br>'physical violence'/exp<br>'torture'/exp<br>'workplace violence'/exp<br>'gun violence'/exp<br>'battered woman'/exp<br>'child abuse survivor'/exp<br>'exposure to violence'/exp<br>'emotional abuse'/exp<br>'elderly abuse'/exp<br>'sexual harassment'/exp<br>'non-sexual harassment'/de<br>'aggression'/de<br>'verbal hostility'/exp<br>'coercion'/exp<br>'intimidation'/exp<br>'dehumanization'/exp<br>'stalking'/de<br>'childhood adversity'/exp<br>'maltreatment'/exp<br>'corporal punishment'/exp<br>'victimization'/exp	'case control study'/exp<br>'crossover procedure'/exp<br>'cohort analysis'/exp<br>'systematic review'/exp<br>'systematic review (topic)'/exp<br>'meta analysis'/exp<br>'meta analysis (topic)'/exp<br>'twin study'/exp	'statistics'/exp<br>'statistical parameters'/exp<br>'risk'/exp<br>'risk ratio'/exp<br>'correlation'/exp<br>'statistical analysis'/exp
Free text terms (searched in title, abstract, and keyword (:ti,ab,kw))	violence<br>'sexual assault'<br>'sexual harassment'<br>'sexual abuse'<br>'sex abuse'<br>rape<br>'forced sex'<br>'sexual coercion'<br>'reproductive coercion'<br>'sex trafficking'<br>'sexual exploitation'<br>'forced marriage*'	'systematic review'<br>meta-analysis<br>cohort<br>cross-over<br>case-control<br>prospective<br>retrospective<br>longitudinal<br>follow-up<br>followup	'risk*'<br>'odds'<br>'cross-product ratio*'<br>'hazards ratio*'<br>'hazard ratio*'<br>statistic*<br>'HR'<br>'RR'<br>'aOR'<br>relation*<br>correlat*<br>associat*

	'child marriage*'<br>'early marriage*'<br>'child bride*'<br>CEFM<br>'female genital mutilation'<br>'female genital cutting'<br>'female circumcision'<br>'female genital circumcision'<br>infibulation*<br>clitoridectom*<br>clitorectom*<br>'ritual female genital surger*'<br>FGM<br>'physical abuse'<br>'psychological abuse'<br>'emotional abuse'<br>'economic abuse'<br>'financial abuse'<br>'verbal abuse'<br>maltreatment<br>'violent discipline'<br>'corporal punishment'<br>'adverse childhood experience*'<br>molestation<br>'child abuse'<br>'partner abuse'<br>'dating abuse'<br>'wife abuse'<br>'spouse abuse'<br>'domestic abuse'<br>'elder abuse'<br>'senior abuse'<br>'aged abuse'<br>victimization<br>dehumanization<br>victimisation<br>dehumanisation<br>stalking<br>cyberviolence<br>cybervictimization<br>cyberstalking		likel*

Supplementary Table 2. Search Terms for CINAHL.

	Concept: Violence	Concept: Study type	Concept: Risk
Subject Headings (MH)	(MH "Sexual Abuse+")<br>(MH "Violence")<br>(MH "Domestic Violence+")<br>(MH "Gender-Based Violence")<br>(MH "Circumcision, Female")<br>(MH "Dating Violence")<br>(MH "Torture")<br>(MH "Workplace Violence")<br>(MH "Gun Violence")<br>(MH "Battered Women")<br>(MH "Child Abuse Survivors")<br>(MH "Exposure to Violence")<br>(MH "Emotional Abuse")<br>(MH "Sexual Harassment")<br>(MH "Aggression")<br>(MH "Verbal Abuse")<br>(MH "Coercion")<br>(MH "Dehumanization")<br>(MH "Stalking")<br>(MH "Adverse Childhood Experiences")<br>(MH "Sibling Violence")<br>(MH "School Violence")<br>(MH "Student Abuse")	(MH "Case Control Studies+")<br>(MH "Crossover Design")<br>(MH "Systematic Review")<br>(MH "Meta Analysis")<br>(MH "Prospective Studies+")<br>(MH "Retrospective Design")	(MH "Statistics+")<br>(MH "Data Analysis, Statistical+")
Free text terms (searched in Title & Abstract; keyword not a searchable field)	violence<br>"sexual assault"<br>"sexual harassment"<br>"sexual abuse"<br>"sex abuse"<br>rape<br>"forced sex"<br>"sexual coercion"<br>"reproductive coercion"<br>"sex trafficking"<br>"sexual exploitation"<br>"forced marriage*"<br>"child marriage*"	"systematic review"<br>meta-analysis<br>cohort<br>cross-over<br>case-control<br>prospective<br>retrospective<br>longitudinal<br>follow-up<br>followup	risk*<br>odds<br>"cross-product ratio*"<br>"hazards ratio*"<br>"hazard ratio*"<br>statistic*<br>"HR"<br>"RR"<br>"aOR"<br>relation*<br>correlat*<br>associat*<br>likel*

	"early marriage*"<br>"child bride*"<br>CEFM<br>"female genital mutilation"<br>"female genital cutting"<br>"female circumcision"<br>"female genital circumcision"<br>infibulation*<br>clitoridectom*<br>clitorectom*<br>"ritual female genital surger*"<br>FGM<br>"physical abuse"<br>"psychological abuse"<br>"emotional abuse"<br>"economic abuse"<br>"financial abuse"<br>"verbal abuse"<br>maltreatment<br>"violent discipline"<br>"corporal punishment"<br>"adverse childhood experience*"<br>molestation<br>"child abuse"<br>"partner abuse"<br>"dating abuse"<br>"wife abuse"<br>"spouse abuse"<br>"domestic abuse"<br>"elder abuse"<br>"senior abuse"<br>"aged abuse"<br>victimization<br>dehumanization<br>victimisation<br>dehumanisation<br>stalking<br>cyberviolence<br>cybervictimization<br>cyberstalking		

Supplementary Table 3. Search Terms for PsycInfo.

	Concept: Violence	Concept: Study type	Concept: Risk
PsycInfo Subject Headings (DE)	DE(<br>"Sexual Violence"<br>"Sex Offenses"<br>"Sexual Abuse"<br>"Sexual Coercion"<br>"Sex Trafficking"<br>"Violence"<br>"Domestic Violence"<br>"Child Abuse"<br>"Elder Abuse"<br>"Circumcision"<br>"Intimate Partner Violence"<br>"Dating Violence"<br>"Physical Abuse"<br>"Physical Discipline"<br>"Punishment"<br>"Rape"<br>"Acquaintance Rape"<br>"Torture"<br>"Workplace Violence"<br>"Gun Violence"<br>"Battered Females"<br>"Exposure to Violence"<br>"Emotional Abuse"<br>"Sexual Harassment"<br>"Aggressive Behavior"<br>"Verbal Abuse"<br>"Coercion"<br>"Stalking"<br>"Childhood Adversity"<br>"School Violence"<br>"Police Violence"<br>"Victimization"<br>)	DE(<br>"Cohort Analysis"<br>"Followup Studies"<br>"Longitudinal Studies"<br>"Retrospective Studies"<br>"Prospective Studies"<br>"Systematic Review"<br>"Meta Analysis"<br>)	DE(<br>"Statistics"<br>"Statistical Analysis"<br>"Risk Assessment"<br>"Statistical Correlation"<br>)
MeSH terms (MA)	MA(<br>"Sex Offenses"<br>"Domestic Violence"<br>"Gender-Based Violence"<br>"Intimate Partner Violence"<br>"Physical Abuse"<br>"Rape"	MA(<br>"Case-Control Studies"<br>"Cross-Over Studies"<br>"Cohort Studies"<br>)	MA(<br>"Statistics as Topic"<br>Risk<br>Odds Ratio<br>)

	"Torture"<br>"Workplace Violence"<br>"Gun violence"<br>"Battered Women"<br>"Adult Survivors of Child abuse"<br>"Exposure to Violence"<br>"Emotional Abuse"<br>"Sexual Harassment"<br>"Coercion"<br>"Dehumanization"<br>"stalking"<br>"adverse childhood experiences"<br>)		
Free text terms (searched in Title, Abstract, and Keywords)	violence<br>"sexual assault"<br>"sexual harassment"<br>"sexual abuse"<br>"sex abuse"<br>rape<br>"forced sex"<br>"sexual coercion"<br>"reproductive coercion"<br>"sex trafficking"<br>"sexual exploitation"<br>"forced marriage*"<br>"child marriage*"<br>"early marriage*"<br>"child bride*"<br>CEFM<br>"female genital mutilation"<br>"female genital cutting"<br>"female circumcision"<br>"female genital circumcision"<br>infibulation*<br>clitoridectom*<br>clitorectom*<br>"ritual female genital surger*"<br>FGM<br>"physical abuse"<br>"psychological abuse"<br>"emotional abuse"<br>"economic abuse"	"systematic review"<br>meta-analysis<br>cohort<br>cross-over<br>case-control<br>prospective<br>retrospective<br>longitudinal<br>follow-up<br>followup	risk*<br>odds<br>"cross-product ratio*"<br>"hazards ratio*"<br>"hazard ratio*"<br>statistic*<br>"HR"<br>"RR"<br>"aOR"<br>relation*<br>correlat*<br>associat*<br>likel*

	"financial abuse"<br>"verbal abuse"<br>maltreatment<br>"violent discipline"<br>"corporal punishment"<br>"adverse childhood experience*"<br>molestation<br>"child abuse"<br>"partner abuse"<br>"dating abuse"<br>"wife abuse"<br>"spouse abuse"<br>"domestic abuse"<br>"elder abuse"<br>"senior abuse"<br>"aged abuse"<br>victimization<br>dehumanization<br>victimisation<br>dehumanisation<br>stalking<br>cyberviolence<br>cybervictimization<br>cyberstalking		

Supplementary Table 4. Search Terms for Global Index Medicus.

	Concept: Violence	Concept: Study type	Concept: Risk
Subject Descriptors	I01.198.240.748*<br>Violence<br>I01.198.240.856.350*<br>I01.198.240.856.463<br>I01.198.240.856.575*<br>I01.198.240.856.688<br>I01.198.240.748.640<br>I01.198.240.856.825<br>I01.198.240.856.912<br>I01.198.240.856.519<br>M01.975.155<br>M01.135.500<br>I01.880.735.900.869<br>I01.880.735.305<br>SP9.020.800.010<br>"Harassment, Non-Sexual"<br>"Aggression"<br>I01.880.604.316<br>Dehumanization<br>F01.145.813.191*<br>I01.880.735.223.500<br>I01.880.735.035	E05.318.372.500.500*<br>E05.318.370.150<br>E05.318.372.500.750*<br>V03.850<br>L01.178.682.759.575<br>V03.600<br>E05.318.370.500*<br>V03.900	E05.318.740*<br>E05.318.740.600.800*<br>E05.318.740.600.600
Title, abstract, subject (tw:())	violence<br>"sexual assault"<br>"sexual harassment"<br>"sexual abuse"<br>"sex abuse"<br>rape<br>"forced sex"<br>"sexual coercion"<br>"reproductive coercion"<br>"sex trafficking"<br>"sexual exploitation"<br>"forced marriage"<br>"forced marriages"<br>"child marriage"<br>"child marriages"<br>"early marriage"<br>"early marriages"<br>"child bride"<br>"child brides"<br>CEFM<br>"female genital mutilation"<br>"female genital cutting"	"systematic review"<br>"meta-analysis"<br>cohort<br>"cross-over"<br>"case-control"<br>Prospective<br>retrospective<br>longitudinal<br>"follow-up"<br>followup	risk*<br>odds<br>"cross-product ratio"<br>"cross-product ratios"<br>"hazards ratio"<br>"hazards ratios"<br>"hazard ratio"<br>"hazard ratios"<br>statistic*<br>"HR"<br>"RR"<br>"aOR"<br>relation*<br>correlat*<br>associat*<br>likel*

	"female circumcision"<br>"female genital circumcision"<br>infibulation*<br>clitoridectom*<br>clitorectom*<br>"ritual female genital surgery"<br>"ritual female genital surgeries"<br>FGM<br>"physical abuse"<br>"psychological abuse"<br>"emotional abuse"<br>"economic abuse"<br>"financial abuse"<br>"verbal abuse"<br>maltreatment<br>"violent discipline"<br>"corporal punishment"<br>"adverse childhood experience"<br>"adverse childhood experiences"<br>molestation<br>"child abuse"<br>"partner abuse"<br>"dating abuse"<br>"wife abuse"<br>"spouse abuse"<br>"domestic abuse"<br>"elder abuse"<br>"senior abuse"<br>"aged abuse"<br>victimization<br>dehumanization<br>victimisation<br>dehumanisation<br>stalking<br>cyberviolence<br>cybervictimization<br>cyberstalking		

Supplementary Table 5. Search Terms for Cochrane.

	Concept: Violence	Concept: Study type	Concept: Risk
Subject Headings [mh].	[mh "Sex Offenses"].<br>[mh ^"Violence"].<br>[mh "Domestic Violence"].<br>[mh "Gender-Based Violence"].<br>[mh "Intimate Partner Violence"].<br>[mh "Physical Abuse"].<br>[mh "Rape"].<br>[mh "Torture"].<br>[mh "Workplace Violence"].<br>[mh "Gun violence"].<br>[mh "Battered Women"].<br>[mh "Adult Survivors of Child abuse"].<br>[mh "Exposure to Violence"].<br>[mh "Emotional Abuse"].<br>[mh "Sexual Harassment"].<br>[mh ^"Harassment, Non-Sexual"].<br>[mh ^"Aggression"].<br>[mh "Coercion"].<br>[mh "Dehumanization"].<br>[mh "stalking"].<br>[mh "adverse childhood experiences"].	[mh "Case-Control Studies"].<br>[mh "Cross-Over Studies"].<br>[mh "Cohort Studies"].<br>[mh "Systematic Review"].<br>[mh "Meta-Analysis"].<br>[mh "Twin Study"].	[mh "Statistics as Topic"].<br>[mh Risk].<br>[mh "Odds Ratio"].
Free text terms :ti,ab,kw	violence<br>"sexual assault"<br>"sexual harassment"<br>"sexual abuse"<br>"sex abuse"<br>rape<br>"forced sex"<br>"sexual coercion"<br>"reproductive coercion"<br>"sex trafficking"<br>"sexual exploitation"<br>((forced OR child OR early) NEXT marriage*)<br>(child NEXT bride*)<br>CEFM<br>"female genital mutilation"<br>"female genital cutting"	"systematic review"<br>"meta-analysis"<br>"cohort"<br>"cross-over"<br>"case-control"<br>"prospective"<br>"retrospective"<br>"longitudinal"<br>"follow-up"<br>"followup"	"risk"<br>"odds"<br>"cross-product ratio"<br>"cross-product ratios"<br>"hazards ratio"<br>"hazards ratios"<br>"hazard ratio"<br>"hazard ratios"<br>statistic*<br>"HR"<br>"RR"<br>"aOR"<br>relation*<br>correlat*<br>associat*<br>likel*

	"female circumcision"<br>"female genital circumcision"<br>infibulation*<br>clitoridectom*<br>clitorectom*<br>"ritual female genital surgery"<br>"ritual female genital surgeries"<br>FGM<br>((physical OR psychological OR emotional OR economic OR financial OR verbal) NEXT abuse)<br>maltreatment<br>"violent discipline"<br>"corporal punishment"<br>"adverse childhood experience"<br>"adverse childhood experiences"<br>molestation<br>((child OR partner OR dating OR wife OR spouse OR domestic OR elder OR senior OR aged) NEXT abuse)<br>victimization<br>dehumanization<br>victimisation<br>dehumanisation<br>stalking<br>cyberviolence<br>cybervictimization<br>cyberstalking		

Supplementary Table 6. Search Terms for Web of Science.

	Concept: Violence	Concept: Study type	Concept: Risk
Topic (searches title, abstract, author keywords, Keywords Plus®)	violence<br>"sexual assault"<br>"sexual harassment"<br>"sexual abuse"<br>"sex abuse"<br>rape<br>"forced sex"<br>"sexual coercion"<br>"reproductive coercion"<br>"sex trafficking"<br>"sexual exploitation"<br>"forced marriage*"<br>"child marriage*"<br>"early marriage*"<br>"child bride*"<br>CEFM<br>"female genital mutilation"<br>"female genital cutting"<br>"female circumcision"<br>"female genital circumcision"<br>infibulation*<br>clitoridectom*<br>clitorectom*<br>"ritual female genital surger*"<br>FGM<br>"physical abuse"<br>"psychological abuse"<br>"emotional abuse"<br>"economic abuse"<br>"financial abuse"<br>"verbal abuse"<br>Maltreatment<br>torture<br>"violent discipline"<br>"corporal punishment"<br>"adverse childhood experience*"<br>molestation<br>"child abuse"<br>"partner abuse"	"systematic review"<br>"meta-analysis"<br>cohort<br>cross-over<br>case-control<br>prospective<br>retrospective<br>longitudinal<br>follow-up<br>followup	"risk*"<br>"odds"<br>"cross-product ratio*"<br>"hazards ratio*"<br>"hazard ratio*"<br>statistic*<br>"HR"<br>"RR"<br>"aOR"<br>relation*<br>correlat*<br>associat*<br>likel*

	"dating abuse"<br>"wife abuse"<br>"battered wom*n"<br>"spouse abuse"<br>"domestic abuse"<br>"elder abuse"<br>"senior abuse"<br>"aged abuse"<br>victimization<br>dehumanization<br>victimisation<br>dehumanisation<br>stalking<br>cyberviolence<br>cybervictimization<br>cyberstalking		
