# Supplementary material for: Estimating the global health impact of gender-based violence and violence against children: a systematic review and meta-analysis protocol
Source: BMJ Open. 2022 Jun 27;12(6):e061248. doi: 10.1136/bmjopen-2022-061248 (PMC9240882; doi:10.1136/bmjopen-2022-061248)
Supplement: Author's manuscript [file bmjopen-2022-061248.draft_revisions.pdf]

BMJ Open is committed to open peer review. As part of this commitment we make the peer review history of every article we publish publicly available.

When an article is published we post the peer reviewers' comments and the authors' responses online. We also post the versions of the paper that were used during peer review. These are the versions that the peer review comments apply to.

The versions of the paper that follow are the versions that were submitted during the peer review process. They are not the versions of record or the final published versions. They should not be cited or distributed as the published version of this manuscript.

BMJ Open is an open access journal and the full, final, typeset and author-corrected version of record of the manuscript is available on our site with no access controls, subscription charges or pay-per-view fees (http://bmjopen.bmj.com).

If you have any questions on BMJ Open's open peer review process please email info.bmjopen@bmj.com

BMJ Open

Estimating the global health impact of gender- and age-based violence: a systematic review and meta-analysis protocol

Journal:	*BMJ Open*
Manuscript ID	bmjopen-2022-061248
Article Type:	Protocol
Date Submitted by the Author:	19-Jan-2022
Complete List of Authors:	Spencer, Cory; Institute for Health Metrics and Evaluation,<br>Baeza, María Jose; University of Miami School of Nursing and Health Studies<br>Chandan, Jaidev Kaur; University of Warwick Warwick Medical School<br>Debure, Alexandra; University of Miami School of Nursing and Health Studies<br>Herbert, Molly; Institute for Health Metrics and Evaluation<br>Jewell, Teresa; University of Washington Health Sciences Library<br>Khalil, Mariam; Institute for Health Metrics and Evaluation<br>Lim, Rachel Qian Hui; Queen Mary University of London Barts and The London School of Medicine and Dentistry<br>Minhas, Sonica; Queen Mary University of London Barts and The London School of Medicine and Dentistry<br>Chandan, Joht Singh; University of Birmingham Institute of Applied Health Research<br>Gakidou, Emmanuela; Institute for Health Metrics and Evaluation<br>Metheny, Nicholas; University of Miami School of Nursing and Health Studies
Keywords:	EPIDEMIOLOGY, PUBLIC HEALTH, STATISTICS & RESEARCH METHODS

SCHOLARONE™
Manuscripts

Estimating the global health impact of gender- and age-based violence: a systematic review and meta-analysis protocol

Cory N Spencer[1], María Jose Baeza[2], Jaidev Kaur Chandan[3], Alexandra Debure[2], Molly Herbert[1], Teresa Jewell[4], Mariam Khalil[1], Rachel Qian Hui Lim[5], Sonica Minhas[5], Joht Singh Chandan[6]*, Emmanuela Gakidou[1]*, Nicholas Metheny[2]*

*Joint senior author

Corresponding author:

Cory N Spencer
Institute for Health Metrics and Evaluation
University of Washington
Population Health Building/Hans Rosling Center
3980 15th Ave. NE, Seattle, WA 98195 USA
UW Campus Box #351615
781-249-0364
corycns@uw.edu

Affiliations:

[1]Institute for Health Metrics and Evaluation, University of Washington, Seattle, WA, USA
[2]School of Nursing and Health Studies, University of Miami, Coral Gables, FL, USA
[3]Warwick Medical School, University of Warwick, Coventry, UK
[4]Health Sciences Library, University of Washington, Seattle, WA, USA
[5]Barts and the London School of Medicine and Dentistry, Queen Mary's University of London, London, UK
[6]Institute of Applied Health Research, University of Birmingham, Birmingham, UK

Word Count: 3,295

For peer review only - http://bmjopen.bmj.com/site/about/guidelines.xhtml

Abstract

Introduction: Exposure to gender- and age-based violence can result in substantial morbidity and mortality. Previous reviews of health outcomes associated with gender- or age-based violence have focused on limited definitions of exposure to violence (i.e., physical and sexual intimate partner violence; childhood maltreatment) and often investigate associations only with predefined health outcomes. Despite evidence that exposures to violence may lead to negative impacts that compound over time, few previous efforts have incorporated a life course approach. In this protocol, we describe a systematic review and meta-analysis for a comprehensive assessment of the impact of violence exposure on health outcomes and health-related risk factors across the life course.

Methods and analysis: Electronic databases will be searched from 1st January 1970 to 30th September 2021 and searches updated to the current date prior to final preparation of results. Two independent reviewers will screen each article, and data will be extracted for estimates of association between violence exposure and health outcomes or health-related risk factors. Study quality will be primarily assessed via the Grading of Recommendations, Assessment, Development and Evaluations risk of bias guidelines. For exposure-outcome pairs with sufficient available data, evidence will be synthesized via a Meta-regression---Bayesian, regularized, trimmed model and confidence in the cumulative evidence assessed via the burden of proof risk function. Where possible, variations in estimates of association by subgroup, i.e. age, sex or gender, will be explored.

Ethics and dissemination: Formal ethical approval is not required. Findings from this review will be used to inform improved estimation of gender- and age-based violence within the Global Burden of Disease Study. The review has been undertaken in conjunction with the Lancet Commission on Gender-based Violence and the Maltreatment of Young People with the aim of providing new data insights for a report on the global response to violence.

Keywords: Epidemiology; public health; gender-based violence; violence against children

For peer review only

For peer review only - http://bmjopen.bmj.com/site/about/guidelines.xhtml

Article Summary

Strengths and limitations of this study

- This review is the first effort to systematically identify and assess all health-related impacts of multiple and overlapping forms of gender and age-based violence.
- Data analysis plans include meta-regression and burden of proof frameworks to synthesize all available evidence and provide policy-direct interpretations of associations.
- Findings from the review will be incorporated in the Global Burden of Disease Study, the most comprehensive observational epidemiological study to date and a critical tool for researchers, advocates, and decision makers.
- Challenges remain in the comparability of definitions of exposure to gender and age-based violence and availability of high quality data for under-studied forms of violence (i.e. cyberviolence, stalking, elder abuse).

For peer review only

For peer review only - http://bmjopen.bmj.com/site/about/guidelines.xhtml

Background

Gender- and age-based violence (including but not limited to all forms gender-based violence (GBV), violence against children (VAC), elder abuse, and violence against women (VAW)) are global public health issues associated with a substantial burden of morbidity and mortality. The immediate impacts lead to physical injuries and death, however the medium and longer term consequences go on to span a variety of physical, mental, sexual, and reproductive health issues [1,2].

The Global Burden of Diseases (GBD), Injuries, and Risk Factors Study has quantified the global disease and disability burden of two forms of gender and aged-based violence risk factors, intimate partner violence (IPV) and childhood sexual abuse (CSA), within a comparative risk assessment framework since 2010 [3–6]. An advantage of the comparative risk assessment framework is the ability to compare the relative contribution to disease and disability among several health risk factors. Indeed, country- and age-specific findings from the GBD have shown IPV to account for more overall disability-adjusted life years (DALYs) in women ages 15-49 than more traditionally highlighted health risk factors such as smoking [7,8]. On a global scale, IPV was estimated to account for 6.44 million (95% uncertainty interval (UI), 3.55-9.87 million) global DALYs among this group in 2019 while, by comparison, smoking contributed to 4.52 million (95% UI 3.87-5.23 million) global DALYs in the same population in 2019 [4]. Much of the estimated health impact stems from GBD meta-analyses of scientific literature, which have found IPV exposure to be associated with a 54% increased risk of depression and 59% increased risk for HIV infection [4]. Likewise, those exposed to CSA have been estimated to be 2.21 times as likely to experience alcohol use disorder (relative risk (RR)=2.21, 95% UI=1.15-4.04) and 1.56 times as likely to experience depression (RR=1.56, 95% UI=1.30-1.86), accounting for 3.67 million (95% UI, 1.75-6.56 million) DALYs among males and females of all ages in 2019 [4]. Despite these findings, the data quality on IPV and CSA outcome pairs are limited by breadth of health outcomes explored and scarcity of studies informing respective relative risk meta-analyses. A more complete understanding of the adverse health outcomes associated with exposure to violence, including definitions beyond those captured in the current GBD framework, and the magnitude of these associations is needed to capture the full scope of gender- and age-based violence and holistically address its negative societal impacts.

Beyond the estimates provided by the internationally comparative GBD studies, existing reviews assessing the health impacts of gender and age-based violence have typically focused on targeted risk-outcome relationships, defining violence and health outcomes within a narrow spectrum and most often exploring outcomes related to GBV and VAC. For example, in 2013, the World Health Organization (WHO), London School of Hygiene and Tropical Medicine, and South African Medical Research Council conducted a systematic review and meta-analysis of a variety of health effects related to specific forms of GBV, measured as physical and/or sexual IPV or non-partner sexual violence (NPSV) [1]. Across studies identified, women exposed to IPV were 1.5 times as likely to become infected with HIV/AIDS and 1.97 times as likely to

For peer review only - http://bmjopen.bmj.com/site/about/guidelines.xhtml

experience depression, among other adverse health outcomes [1]. A lack of comparable studies prevented meta-analysis for NPSV [1]. Following the publication of 2013 report, the World Health Organization curated an extensive database of studies describing the literature explaining the relationship between violence against women (VAW) and VAC with subsequent health outcomes [9]. However, the database is not regularly maintained and has not updated summaries of high-quality evidence on the health impacts of GBV (VAW/IPV) since 2013, which is urgently needed to inform global health policy. In 2018, Bacchus et al. additionally reviewed cohort studies that reported on all health outcomes and behaviors related to recent physical and sexual IPV exposure, finding evidence of a positive, bidirectional relationship between these types of IPV and depressive symptoms [10]. However, there are fewer reviews investigating exposure types beyond physical and sexual IPV (e.g. psychological violence, coercive control, financial abuse, stalking), and those available often define even finer scopes by investigating relationships between narrowly defined forms of violence and narrowly defined health outcomes, including mental health and gynecological morbidity [11,12].

Similar to GBV, there have been attempts to synthesize the literature exploring the breadth of consequences following exposure to VAC and highlighting the relationship between exposure to childhood maltreatment (including CSA) and a wide variety of psychosocial and health outcomes [13,14]. Research into the consequences of VAC has more recently overlapped with the burgeoning literature base describing health outcomes secondary to adverse childhood experience (ACE) exposure, two of which include direct exposure to violence and the witnessing of parental IPV [15]. A recent comprehensive review highlighted the pervasive harms that ACEs place on health throughout the life course [16] and in a secondary analysis found that within Europe and North America, a 10% reduction in ACE prevalence could equate to annual savings of 3 million DALYs or $105 billion [17]. However, despite these efforts to capture the literature on VAC through either exploring childhood maltreatment, CSA, or ACEs as the marker of exposure, included studies are often limited to exposure in high income countries and exclude other forms of violence such as female genital mutilation, trafficking, forced marriage and cyberviolence.

While the existing literature has illuminated the significant health impacts of violence, critical evidence gaps remain. These include the quantification of the health burden of less-studied forms of gender and age-based violence, their outcomes in lower and middle income settings, as well as the importance of removing artificial barriers which have created isolated streams of research. As highlighted through the reviews cited above, the current research trajectory often creates distinctions between GBV/VAC and other forms of life course violence such as elder-abuse [18]. However, considering that GBV and VAC share risk factors, co-occur and can lead to compounding consequences across the life course, there is a clear need to examine gender- and age-based violence in unison. To address these challenges, the Sexual Violence Research Initiative (SVRI), UNICEF Innocenti, and WHO have recently developed a framework of guiding principles encouraging interaction of research in the field of violence epidemiology which we aim to adopt in this proposed work [19].

For peer review only - http://bmjopen.bmj.com/site/about/guidelines.xhtml

To aid in this global effort, we present a systematic review and meta-analysis protocol to generate estimates of a comprehensive range of health impacts associated with exposure to gender- and age-based violence. Findings will contribute to the assessment of risk-outcome relationships and attributable burdens of disease within the GBD. To our knowledge, there has been no other systematic review and meta-analysis conducted with such a life course approach across multiple types of violence.

Methods and analysis

The presentation of our review design follows the Preferred Reporting Items for Systematic review and Meta-Analysis Protocols (PRISMA-P) guidelines (Supplemental File 1) [20].

Aims of the review

The aim of this review is to identify and synthesize all available data on the health impacts of exposure to any form of gender- and age-based violence. This data can then be used to assess risk-outcome relationships and quantify their contribution to global disease and disability burdens.

Specific review questions

1. How does exposure to gender- and age-based violence impact health across the life course?
2. What is the strength of evidence on the associations between exposure to gender- and age-based violence and different health impacts?
3. Do estimates of association vary by characteristics of the violence, global region, characteristics of the perpetrator, and/or characteristics of the victim?

Definitions

Definitions of gender and aged-based violence

We include in our searches the following terms related to gender- and aged-based violence:

- Gender-based violence;
- Intimate partner violence, partner abuse/violence, wife/spouse abuse, dating abuse/violence;
- Sexual abuse, rape, forced sex, sexual assault, sexual coercion, sexual exploitation;
- Reproductive coercion;
- Female genital mutilation, female genital cutting, female circumcision;
- Sex trafficking, forced marriage;
- Physical abuse;
- Psychological abuse, emotional abuse, verbal abuse;
- Economic abuse;
- Cyberviolence, cybervictimization;
- Domestic violence/abuse;
- Adverse childhood experiences that include direct exposure to and witnessing of violence;

For peer review only - http://bmjopen.bmj.com/site/about/guidelines.xhtml

- Child maltreatment, molestation, child abuse;
- Elder abuse, senior abuse, aged abuse;
- Stalking, cyberstalking;
- Dehumanization, torture;
- Workplace violence, student abuse, sexual harassment;
- Gender-based violence perpetrated with a firearm.

We expect author definitions and methods used to measure exposure to vary and will accept all definitions, documenting study definitions and measurement techniques as a part of study-level quality assessment.

Health outcomes

We did not restrict searches to pre-defined health outcomes and aim to accept all literature reporting an association between violence exposure and health. Definitions of health outcomes and health-related risk factors will be guided by causes, injuries, and risk factor case definitions from the Global Burden of Diseases study [4,21] and differences in measurement methods or case definitions documented as well. Final selection of associations to be synthesized will depend on the availability of studies that examine the association between a comparable form of exposure and reported health outcome.

Criteria for considering studies for this review

Inclusion

- Study design: Case-control, cohort, or case-crossover studies.
- Participants: Studies conducted in participant groups likely to be generalizable to the population of interest. Exposed groups are defined as any individual who has experienced a form of gender- or age-based violence throughout the lifetime. Comparators will be non-exposed control groups, or study groups without reported exposure to a form of gender- or age-based violence.
- Outcomes: Studies reporting an estimate of association (either relative risk, risk ratio, odds-ratio, hazard ratio, or similar) or reporting cases and non-cases among those exposed and unexposed. If not provided directly, studies providing enough information to allow an estimate of relative to risk to be calculated will meet inclusion criteria.

Exclusion

- Study design: Cross-sectional, ecological, case series or case studies. We do not anticipate there to be any experimental studies, however, these will also be excluded.
- Participants: Studies conducted in subgroups identified only by convenience sampling or subgroups identified via a shared characteristic that is likely related to risk of exposure to violence or the reported health outcome (e.g. domestic violence shelter residents).
- Exposure measurement: Studies that report only an aggregate measure of exposure combining exposure to a form of violence with other, non-eligible exposures (e.g., reports a composite adverse childhood experience score only) will be excluded.

For peer review only - http://bmjopen.bmj.com/site/about/guidelines.xhtml

- Does not meet minimum reporting criteria: Studies missing essential data, i.e., do not report effect sizes and uncertainty information (confidence intervals, sample sizes) or the data needed to impute an effect size with uncertainty information.
- Studies reporting on the same exposure and outcome using the same data: The study with the longest follow-up time period or most complete dataset will be included.

Search strategy for identifying relevant studies

PubMed, Embase, CINAHL, PsycInfo, Global Index Medicus, Cochrane, and Web of Science were searched using controlled vocabulary search terms. All relevant studies published between January 1st 1970 and September 30th 2021 will be considered, regardless of language of publication or study setting. Immediately prior to preparing final results from the review and meta-analysis, searches will be updated to the current month to retrieve for inclusion any further studies identified. The search strategy for PubMed is provided in Table 1. The search terms for Embase, CINAHL, PsycInfo, Global Index Medicus, Cochrane, and Web of Science are provided in the Supplementary Tables 1, 2, 3, 4, 5, and 6, respectively (Supplemental File 2).

For peer review only

For peer review only - http://bmjopen.bmj.com/site/about/guidelines.xhtml

Table 1. Search Terms and Strategy for PubMed.

Search Terms	Concept
1. "Sex Offenses"[mh].<br>2. "Violence"[mh:noexp].<br>3. "Domestic Violence"[mh].<br>4. "Gender-Based Violence"[mh].<br>5. "Intimate Partner Violence"[mh].<br>6. "Physical Abuse"[mh].<br>7. "Rape"[mh].<br>8. "Torture"[mh].<br>9. "Workplace Violence"[mh].<br>10. "Gun violence"[mh].<br>11. "Battered Women"[mh].<br>12. "Adult Survivors of Child abuse"[mh].<br>13. "Exposure to Violence"[mh].<br>14. "Emotional Abuse"[mh].<br>15. "Sexual Harassment"[mh].<br>16. "Harassment, Non-Sexual"[mh:noexp].<br>17. "Emotional abuse"[mh].<br>18. "Aggression"[mh:noexp].<br>19. "Dehumanization"[mh].<br>20. "stalking"[mh].<br>21. “adverse childhood experiences”[mh].<br>22. violence[tiab].<br>23. "sexual assault"[tiab].<br>24. "sexual harassment"[tiab].<br>25. “sexual abuse”[tiab].<br>26. “sex abuse”[tiab].<br>27. rape[tiab].<br>28. “forced sex”[tiab].<br>29. “sexual coercion”[tiab].<br>30. “reproductive coercion”[tiab].<br>31. “sex trafficking”[tiab].<br>32. “sexual exploitation”[tiab].<br>33. “forced marriage”[tiab].<br>34. “female genital mutilation”[tiab].<br>35. “female genital cutting”[tiab].<br>36. “female circumcision”[tiab].<br>37. “female genital circumcision”[tiab].<br>38. “physical abuse”[tiab].<br>39. “psychological abuse”[tiab].<br>40. “emotional abuse”[tiab].<br>41. “economic abuse”[tiab].<br>42. "verbal abuse"[tiab].<br>43. maltreatment[tiab].<br>44. “violent discipline”[tiab].<br>45. “corporal punishment”[tiab].<br>46. “adverse childhood experience*”[tiab].<br>47. molestation[tiab].<br>48. “child abuse”[tiab].<br>49. “partner abuse”[tiab].<br>50. "dating abuse"[tiab].	Violence exposure

For peer review only - http://bmjopen.bmj.com/site/about/guidelines.xhtml

51. “wife abuse”[tiab].<br>52. “spouse abuse”[tiab].<br>53. “domestic abuse”[tiab].<br>54. “elder abuse”[tiab].<br>55. “senior abuse”[tiab].<br>56. “aged abuse”[tiab].<br>57. victimization[tiab].<br>58. dehumanization[tiab].<br>59. victimisation[tiab].<br>60. dehumanisation[tiab].<br>61. stalking[tiab].<br>62. cyberviolence[tiab].<br>63. cybervictimization[tiab].<br>64. cyberstalking[tiab].<br>65. Or/1-64	
66. Case-Control Studies[mh].<br>67. Cross-Over Studies[mh].<br>68. Cohort Studies[mh].<br>69. Systematic Review[pt].<br>70. Meta-Analysis[pt].<br>71. "Twin Study"[pt].<br>72. “systematic review”[tiab].<br>73. “meta-analysis”[tiab].<br>74. “cohort”[tiab].<br>75. “cross-over”[tiab].<br>76. “case-control”[tiab].<br>77. “prospective”[tiab].<br>78. “retrospective”[tiab].<br>79. “longitudinal”[tiab].<br>80. “follow-up”[tiab].<br>81. “followup”[tiab].<br>82. Or/66-81	Study type
83. "Statistics as Topic"[mh].<br>84. Risk[mh].<br>85. Odds Ratio[mh].<br>86. “risk*”[tiab].<br>87. “odds”[tiab].<br>88. “cross-product ratio*”[tiab].<br>89. “hazards ratio*”[tiab].<br>90. “hazard ratio*”[tiab].<br>91. statistic*[tiab].<br>92. “HR”[tiab].<br>93. “RR”[tiab].<br>94. “aOR”[tiab].<br>95. relation*[tiab].<br>96. correlat*[tiab].<br>97. associat*[tiab].<br>98. Or/83-97	Risk
99. "1970/01/01"[PDat]. : "2021/09/30"[PDat].	Date restriction – all available literature since 1970
100.65 AND 82 AND 98 AND 99	

For peer review only - http://bmjopen.bmj.com/site/about/guidelines.xhtml

Data management and extraction

Search results will be merged and duplicates removed using the systematic review management software Covidence [22]. All reviewers will initially screen the first 50 search results and meet to compare screening decisions and clarify any questions with regard to the inclusion criteria. Two independent reviewers will screen each article, and JSC, NM and CS will review and resolve all conflicts that arise during screening decisions. Non-English publications will be reviewed using the language fluencies (Spanish, French, and Portuguese) of the reviewers.

Studies that meet inclusion criteria will additionally be full-text screened and excluded if found to meet any of the exclusion criteria. For full text review and extraction, 10% of articles will be reviewed by two independent reviewers and a meeting held to resolve misunderstandings and ensure all reviewers clearly understand inclusion and exclusion criteria. The remaining 90% of articles will be reviewed by one independent reviewer. If reviewers are unable to access the full text of a publication, the reviewers will reach out directly to the corresponding author and wait a maximum of one month for response, after which point the article will be excluded.

Reviewers will extract data using a standardized form created in Covidence [22]. The data extraction form will include variables related to (1) characteristics of the study, (2) characteristics of the study population, (3) exposure and outcome measurement, (4) effect size and uncertainty, (5) risk of bias (quality assessment) [23,24]. Data items are provided in Table 2.

For peer review only

Table 2. Variables to be collected in the data extraction process.

Category	Data items
1. Study characteristics	- author(s)<br>- citation<br>- year of publication<br>- geographical location<br>- study year(s)<br>- study design<br>- study name
2. Population characteristics	- selection criteria<br>- age summary statistics (range or median and standard deviation)<br>- sex or gender summary statistics (percent women or percent female)<br>- follow-up duration
3. Exposure and outcome measurement	- exposure definition<br>- exposure assessment frequency<br>- exposure recall period<br>- exposure type included in relative risk estimation<br>- exposure categories<br>- outcome definition
4. Effect size and uncertainty	- effect size measure (e.g., relative risk, odds ratio, incidence rate ratio, hazard ratio)<br>- effect size<br>- confidence interval and level<br>- non-confidence interval uncertainty type and value<br>- sample size (total, exposed, unexposed)<br>- person-time (total, exposed, unexposed)<br>- number of events (total, among exposed, among unexposed)<br>- whether main or subgroup analysis<br>- description of subgroup analysis
5. Risk of bias (quality assessment)	- exposure assessment method<br>- exposure assessment instrument<br>- outcome assessment method<br>- outcome assessment instrument<br>- representativeness<br>- confounders adjusted for in reported effect size<br>- percent of participants for which exposure data was obtained (for case-control studies)<br>- drop-out rate (for cohort studies)<br>- risk of reverse causation

For peer review only - http://bmjopen.bmj.com/site/about/guidelines.xhtml

If a study reports on multiple forms of violence exposure, multiple associated health outcomes, or reports findings by subgroup or model specification, data pertaining to each sub-analysis will be extracted in addition to any aggregate results. In the case of a study reporting effect sizes for multiple model specifications, the most appropriately adjusted model will be selected for inclusion in meta-analysis.

Risk of bias in individual studies

Sources of bias will be assessed and collected during data extraction. Following the Grading of Recommendations, Assessment, Development and Evaluations (GRADE) approach [24], risk of bias criteria include,

- Exposure measurement: How exposure to violence was assessed (whether standard, acts-based and specific questions were asked, e.g. "Have you ever been shoved, slapped, hit, or kicked by an intimate partner?" versus questions that rely upon participants' own definition of abuse, e.g. "Have you ever experienced domestic abuse?"). In addition, whether exposure was based upon self-reports or another source (e.g. health records).
- Outcome measurement: How reported health outcome(s) were measured (by physician diagnosis, diagnostic survey instruments, electronic health records, or reported as symptom severity or symptom scales).
- Representativeness of study population: If a study sample was based on the general population or if study results are reported from a sub-group for which there are prior reasons to believe that findings would be different.
- Control for confounding: If a study statistically controlled for confounding using all major known confounders, including age, sex, education, income, and other critical determinants of the health outcome.
- Selection bias: If a study is at risk of selection bias, based upon percent follow-up for longitudinal study designs and based upon the percentages of cases and controls for which exposure data can be ascertained for case-control designs.
- Reverse causation: If a study is at risk of reverse causation, evaluated through study design and opportunity for recall bias (i.e. case-control studies) [25].

Data Synthesis

If there are at least 3 studies identified with a comparable form of exposure and reported health outcome, we will synthesize effect sizes using a Meta-regression---Bayesian, regularized, trimmed (MR-BRT) model [4,26].

For each risk-outcome pair identified, we will use the MR-BRT tool to perform a meta-regression analysis of the risk of the given outcome for those exposed to the violence type relative to the reference category of those not exposed to the violence type. For risk-outcome pairs with sufficient data points, we will introduce likelihood-based trimming to detect and remove outliers before fitting the model, with an inlier fraction of 90% [26].

For peer review only - http://bmjopen.bmj.com/site/about/guidelines.xhtml

For each risk-outcome pair meta-analysis, we will consider study-level covariates with the potential to bias the study's reported effect size estimates and adjust for these covariates if they are found to significantly bias the estimated relative risk. The MR-BRT tool includes an automated covariate selection process using a Lasso strategy to identify statistically significant covariates at a significance threshold of 0.05 [26,27].

The MR-BRT tool quantifies between-study heterogeneity by accounting for heterogeneity uncertainty and small numbers of studies [26]. In this approach, the Fisher information matrix is used to estimate uncertainty of the between-study heterogeneity parameter, γ [26,28]. The final uncertainty estimate reflects both the posterior uncertainty corresponding to the fixed effect and the 95% quantile of γ, which is sensitive to the number of studies, study design, and reported uncertainty of the effect size [26].

For each risk-outcome pair, we will additionally test for and report publication bias in the input data based upon the Egger's Regression strategy, which tests the degree to which standard error is correlated with effect size in the data, and present funnel plots [29,30].

Additional Analyses

If meta-analysis is not possible with all studies, we will synthesize the included study findings graphically [31] following the systematic review without meta-analysis [SWiM] guidance. This will include forest plots, which will graphically depict all study effect estimates using a single metric (e.g., percent change) for each available health outcome and type of violence [32]. To produce the forest plots, we will transform effect estimates to a comparable metric wherever possible (i.e., where the necessary data are available in the paper or from the authors). Harvest plots will demonstrate where inequities based on, for example, age of exposure, low- and middle-income country vs. high-income country, gender identity, ethnicity/race, sexual orientation, urban-rural location, exist in the available data [33]. When the necessary data are missing, all study effect estimates will be summarized in supplementary tables and discussed as relevant in text. If the necessary data are available (standardized effect estimate, p-value) we will also consider albatross plots to summarize results [34].

Confidence in cumulative evidence

Confidence in risk-outcome pair results will be assessed via the burden of proof risk function (BPRF). For binary risk-outcome pairs, the BPRF is defined as the 5th quantile for the effect size (that includes the effect size uncertainty and the 95% quantile of between-study heterogeneity) closest to the null (log relative risk of 0) and can be interpreted as the smallest effect consistent with available evidence [35]. A risk-outcome score (ROS) and corresponding star-rating will be derived from the BPRF. Star-rating categories are a policy-direct way to interpret the evidence for risk-outcome pairs, with higher star-ratings indicating stronger evidence of an association [35].

For peer review only

For peer review only - http://bmjopen.bmj.com/site/about/guidelines.xhtml

Patient and public involvement

Patients and/or the public were not involved in the design, or conduct, or reporting, or dissemination plans of this research.

Ethics and Dissemination

The proposed review does not require formal ethical approval. Findings from this review will contribute to GBD estimates of the health impact of gender and age-based violence. The GBD includes data on morbidity and mortality from 1990 to present in 204 countries and territories for 369 diseases and injuries and 87 risk factors [36]. It is the most comprehensive worldwide observational epidemiological study to date and a critical tool used by clinicians, policy makers, and researchers. Review findings will inform the GBD assessment of new risks and/or risk-outcome relationships and revisions to the magnitude of currently included associations. Updated global health estimates of the impact of gender and age-based violence will be highlighted in consequent GBD releases and accompanying capstone publications.

In addition, this review is being conducted in conjunction with the Lancet Commission on Gender-Based Violence and the Maltreatment of Young People [37]. The aim of the Commission is to complete a path breaking report on the global response to violence across the life course, complete with new data insights and concrete policy and research recommendations that are informed by survivors and advocates. This report will be published by The Lancet in an effort to initiate debate, offer insight and explanation, and influence decision makers across the globe regarding gender- and age-based violence.

Author Contributions

The initial PubMed search strategy was developed by CS and refined and adapted to other databases by TJ. The writing and methodological plan for this protocol was developed by CS, JSC, NM, and EG. Further revisions to the protocol were made by all remaining authors (MB, AD, MH, TJ, JKC, MK, RL, and SM) and the final copy of the manuscript was approved by all authors.

Funding statement

This work was supported by the Bill and Melinda Gates foundation grant number INV-018617. The funders of had no role in the review design or the writing of the report.

Competing interests statement

None declared.

Data Availability Statement

No data are available from this protocol.

For peer review only - http://bmjopen.bmj.com/site/about/guidelines.xhtml

References

1 WHO, Department of Reproductive Health and Research, London School of Hygiene and Tropical Medicine, South African Medical Research Council. Global and regional estimates of violence against women: prevalence and health effects of intimate partner violence and non-partner sexual violence.

2 The Lancet. Adversity in childhood: how the past defines the future. *Lancet Lond Engl* 2016;**388**:2324. doi:10.1016/S0140-6736(16)32172-9

3 Lim SS, Vos T, Flaxman AD, *et al.* A comparative risk assessment of burden of disease and injury attributable to 67 risk factors and risk factor clusters in 21 regions, 1990-2010: a systematic analysis for the Global Burden of Disease Study 2010. *Lancet Lond Engl* 2012;**380**:2224–60. doi:10.1016/S0140-6736(12)61766-8

4 GBD 2019 Risk Factors Collaborators. Global burden of 87 risk factors in 204 countries and territories, 1990-2019: a systematic analysis for the Global Burden of Disease Study 2019. *Lancet Lond Engl* 2020;**396**:1223–49. doi:10.1016/S0140-6736(20)30752-2

5 Murray CJ, Lopez AD. Global mortality, disability, and the contribution of risk factors: Global Burden of Disease Study. *Lancet Lond Engl* 1997;**349**:1436–42. doi:10.1016/S0140-6736(96)07495-8

6 Murray CJ, Ezzati M, Lopez AD, *et al.* Comparative quantification of health risks conceptual framework and methodological issues. *Popul Health Metr* 2003;**1**:1. doi:10.1186/1478-7954-1-1

7 Vos T, Astbury J, Piers LS, *et al.* Measuring the impact of intimate partner violence on the health of women in Victoria, Australia. *Bull World Health Organ* 2006;**84**:739–44.

8 Mokdad AH, Forouzanfar MH, Daoud F, *et al.* Global burden of diseases, injuries, and risk factors for young people's health during 1990–2013: a systematic analysis for the Global Burden of Disease Study 2013. *The Lancet* 2016;**387**:2383–401. doi:10.1016/S0140-6736(16)00648-6

9 World Health Organization. Violence Info – A global knowledge platform for preventing violence. http://apps.who.int/violence-info/ (accessed 29 Nov 2021).

10 Bacchus LJ, Ranganathan M, Watts C, *et al.* Recent intimate partner violence against women and health: a systematic review and meta-analysis of cohort studies. *BMJ Open* 2018;**8**:e019995. doi:10.1136/bmjopen-2017-019995

11 Hassam T, Kelso E, Chowdary P, *et al.* Sexual assault as a risk factor for gynaecological morbidity: An exploratory systematic review and meta-analysis. *Eur J Obstet Gynecol Reprod Biol* 2020;**255**:222–30. doi:10.1016/j.ejogrb.2020.10.038

For peer review only - http://bmjopen.bmj.com/site/about/guidelines.xhtml

12 Devries KM, Mak JY, Bacchus LJ, *et al.* Intimate partner violence and incident depressive symptoms and suicide attempts: a systematic review of longitudinal studies. *PLoS Med* 2013;**10**:e1001439. doi:10.1371/journal.pmed.1001439

13 Gilbert R, Widom CS, Browne K, *et al.* Burden and consequences of child maltreatment in high-income countries. *Lancet Lond Engl* 2009;**373**:68–81. doi:10.1016/S0140-6736(08)61706-7

14 Hailes HP, Yu R, Danese A, *et al.* Long-term outcomes of childhood sexual abuse: an umbrella review. *Lancet Psychiatry* 2019;**6**:830–9. doi:10.1016/S2215-0366(19)30286-X

15 Centers for Disease Control and Prevention. Adverse Childhood Experiences (ACEs). 2021.https://www.cdc.gov/violenceprevention/aces/index.html (accessed 9 Dec 2021).

16 Hughes K, Bellis MA, Hardcastle KA, *et al.* The effect of multiple adverse childhood experiences on health: a systematic review and meta-analysis. *Lancet Public Health* 2017;**2**:e356–66. doi:10.1016/S2468-2667(17)30118-4

17 Bellis MA, Hughes K, Ford K, *et al.* Life course health consequences and associated annual costs of adverse childhood experiences across Europe and North America: a systematic review and meta-analysis. *Lancet Public Health* 2019;**4**:e517–28. doi:10.1016/S2468-2667(19)30145-8

18 Guedes A, Bott S, Garcia-Moreno C, *et al.* Bridging the gaps: a global review of intersections of violence against women and violence against children. *Glob Health Action* 2016;**9**:31516. doi:10.3402/gha.v9.31516

19 UNICEF Office of Research-Innocenti. Joining forces to develop a research agenda on intersections of violence against children and violence against women. UNICEF-IRC. https://www.unicef-irc.org/article/2121-joining-forces-to-develop-a-research-agenda-on-intersections-of-violence-against-children-and-violence.html (accessed 29 Nov 2021).

20 Moher D, Shamseer L, Clarke M, *et al.* Preferred reporting items for systematic review and meta-analysis protocols (PRISMA-P) 2015 statement. *Syst Rev* 2015;**4**:1. doi:10.1186/2046-4053-4-1

21 Vos T, Lim SS, Abbafati C, *et al.* Global burden of 369 diseases and injuries in 204 countries and territories, 1990–2019: a systematic analysis for the Global Burden of Disease Study 2019. *The Lancet* 2020;**396**:1204–22. doi:10.1016/S0140-6736(20)30925-9

22 *Covidence systematic review software*. Melbourne, Australia: : Veritas Health Innovation www.covidence.org (accessed 3 Nov 2021).

23 Wells G, Shea B, O'Connel D, *et al.* The Newcastle-Ottawa Scale (NOS) for assessing the quality of nonrandomised studies in meta-analyses.Ottawa Hospital Research Institute. Published Online First:

For peer review only - http://bmjopen.bmj.com/site/about/guidelines.xhtml

2013.http://www.ohri.ca/programs/clinical_epidemiology/oxford.asp, (accessed 29 Nov 2021).

24 Guyatt GH, Oxman AD, Vist G, *et al.* GRADE guidelines: 4. Rating the quality of evidence--study limitations (risk of bias). *J Clin Epidemiol* 2011;**64**:407–15. doi:10.1016/j.jclinepi.2010.07.017

25 Chouinard E, Walter S. Recall bias in case-control studies: an empirical analysis and theoretical framework. *J Clin Epidemiol* 1995;**48**:245–54. doi:10.1016/0895-4356(94)00132-a

26 Zheng P, Barber R, Sorensen RJD, *et al.* Trimmed Constrained Mixed Effects Models: Formulations and Algorithms. *J Comput Graph Stat* 2021;**30**:544–56. doi:10.1080/10618600.2020.1868303

27 Tibshirani R. Regression Shrinkage and Selection via the Lasso. *J R Stat Soc Ser B Methodol* 1996;**58**:267–88.

28 Casella G, Berger RL. *Statistical Inference*. Cengage Learning 2021.

29 Egger M, Smith GD, Schneider M, *et al.* Bias in meta-analysis detected by a simple, graphical test. *BMJ* 1997;**315**:629–34. doi:10.1136/bmj.315.7109.629

30 Sterne JAC, Egger M. Regression Methods to Detect Publication and Other Bias in Meta-Analysis. In: *Publication Bias in Meta-Analysis*. John Wiley & Sons, Ltd 2005. 99–110. doi:10.1002/0470870168.ch6

31 Campbell M, McKenzie JE, Sowden A, *et al.* Synthesis without meta-analysis (SWiM) in systematic reviews: reporting guideline. *BMJ* 2020;**368**:l6890. doi:10.1136/bmj.l6890

32 Humphreys DK, Ogilvie D. Synthesising evidence for equity impacts of population-based physical activity interventions: a pilot study. *Int J Behav Nutr Phys Act* 2013;**10**:76. doi:10.1186/1479-5868-10-76

33 Ogilvie D, Fayter D, Petticrew M, *et al.* The harvest plot: A method for synthesising evidence about the differential effects of interventions. *BMC Med Res Methodol* 2008;**8**:8. doi:10.1186/1471-2288-8-8

34 Harrison S, Jones HE, Martin RM, *et al.* The albatross plot: A novel graphical tool for presenting results of diversely reported studies in a systematic review. *Res Synth Methods* 2017;**8**:281–9. doi:10.1002/jrsm.1239

35 GBD 2020 Risk Score Collaborators. Burden of proof risk functions: a meta-analytic approach to assessing evidence. *Nat Med* in review.

For peer review only - http://bmjopen.bmj.com/site/about/guidelines.xhtml

36 About the Global Burden of Disease. https://www.thelancet.com/gbd/about (accessed 29 Nov 2021).

37 Knaul FM, Bustreo F, Horton R. Countering the pandemic of gender-based violence and maltreatment of young people: The Lancet Commission. *The Lancet* 2020;**395**:98–9. doi:10.1016/S0140-6736(19)33136-8

For peer review only

For peer review only - http://bmjopen.bmj.com/site/about/guidelines.xhtml

Reporting checklist for protocol of a systematic review and meta analysis.

Based on the PRISMA-P guidelines.

Instructions to authors

Complete this checklist by entering the page numbers from your manuscript where readers will find each of the items listed below.

Your article may not currently address all the items on the checklist. Please modify your text to include the missing information. If you are certain that an item does not apply, please write "n/a" and provide a short explanation.

Upload your completed checklist as an extra file when you submit to a journal.

In your methods section, say that you used the PRISMA-Preporting guidelines, and cite them as:

Moher D, Shamseer L, Clarke M, Ghersi D, Liberati A, Petticrew M, Shekelle P, Stewart LA. Preferred Reporting Items for Systematic Review and Meta-Analysis Protocols (PRISMA-P) 2015 statement. Syst Rev. 2015;4(1):1.

		Reporting Item	Page Number
**Title**			
Identification	#1a	Identify the report as a protocol of a systematic review	1
Update	#1b	If the protocol is for an update of a previous systematic review, identify as such	n/a

For peer review only

**Registration**			
	#2	If registered, provide the name of the registry (such as PROSPERO) and registration number	n/a. Record currently under consideration for registration with PROSPERO.
**Authors**			
Contact	#3a	Provide name, institutional affiliation, e-mail address of all protocol authors; provide physical mailing address of corresponding author	1
Contribution	#3b	Describe contributions of protocol authors and identify the guarantor of the review	15
**Amendments**			
	#4	If the protocol represents an amendment of a previously completed or published protocol, identify as such and list changes; otherwise, state plan for documenting important protocol amendments	n/a. This protocol is not an amendment.
**Support**			
Sources	#5a	Indicate sources of financial or other support for the review	15
Sponsor	#5b	Provide name for the review funder and / or sponsor	15

For peer review only

Role of sponsor or funder	#5c	Describe roles of funder(s), sponsor(s), and / or institution(s), if any, in developing the protocol	15
**Introduction**			
Rationale	#6	Describe the rationale for the review in the context of what is already known	4-6
Objectives	#7	Provide an explicit statement of the question(s) the review will address with reference to participants, interventions, comparators, and outcomes (PICO)	6
**Methods**			
Eligibility criteria	#8	Specify the study characteristics (such as PICO, study design, setting, time frame) and report characteristics (such as years considered, language, publication status) to be used as criteria for eligibility for the review	7-8
Information sources	#9	Describe all intended information sources (such as electronic databases, contact with study authors, trial registers or other grey literature sources) with planned dates of coverage	8
Search strategy	#10	Present draft of search strategy to be used for at least one electronic database, including planned limits, such that it could be repeated	8-10; S2

For peer review only

Study records - data management	#11a	Describe the mechanism(s) that will be used to manage records and data throughout the review	10-11
Study records - selection process	#11b	State the process that will be used for selecting studies (such as two independent reviewers) through each phase of the review (that is, screening, eligibility and inclusion in meta-analysis)	11
Study records - data collection process	#11c	Describe planned method of extracting data from reports (such as piloting forms, done independently, in duplicate), any processes for obtaining and confirming data from investigators	11-12
Data items	#12	List and define all variables for which data will be sought (such as PICO items, funding sources), any pre-planned data assumptions and simplifications	11-12
Outcomes and prioritization	#13	List and define all outcomes for which data will be sought, including prioritization of main and additional outcomes, with rationale	6-7
Risk of bias in individual studies	#14	Describe anticipated methods for assessing risk of bias of individual studies, including whether this will be done at the outcome or study level, or both; state how this information will be used in data synthesis	12-13

For peer review only

Data synthesis	#15a	Describe criteria under which study data will be quantitatively synthesised	13-14
Data synthesis	#15b	If data are appropriate for quantitative synthesis, describe planned summary measures, methods of handling data and methods of combining data from studies, including any planned exploration of consistency (such as I2, Kendall's τ)	13-14
Data synthesis	#15c	Describe any proposed additional analyses (such as sensitivity or subgroup analyses, meta-regression)	13-14
Data synthesis	#15d	If quantitative synthesis is not appropriate, describe the type of summary planned	13-14
Meta-bias(es)	#16	Specify any planned assessment of meta-bias(es) (such as publication bias across studies, selective reporting within studies)	13
Confidence in cumulative evidence	#17	Describe how the strength of the body of evidence will be assessed (such as GRADE)	14

Notes:

- 2: n/a. Record currently under consideration for registration with PROSPERO.
- 4: n/a. This protocol is not an amendment.

For peer review only

The PRISMA-P elaboration and explanation paper is distributed under the terms of the Creative Commons Attribution License CC-BY. This checklist was completed on 13. January 2022 using https://www.goodreports.org/, a tool made by the EQUATOR Network in collaboration with Penelope.ai

For peer review only

Supplementary Table 1. Search Terms for Embase.

	Concept: Violence	Concept: Study type	Concept: Risk
Subject Headings (Emtree)	'sexual violence'/exp<br>'forced sex'/exp<br>'violence'/de<br>'domestic violence'/exp<br>'gender based violence'/exp<br>'partner violence'/exp<br>'dating violence'/exp<br>'physical abuse'/exp<br>'physical violence'/exp<br>'torture'/exp<br>'workplace violence'/exp<br>'gun violence'/exp<br>'battered woman'/exp<br>'child abuse survivor'/exp<br>'exposure to violence'/exp<br>'emotional abuse'/exp<br>'elderly abuse'/exp<br>'sexual harassment'/exp<br>'non-sexual harassment'/de<br>'aggression'/de<br>'verbal hostility'/exp<br>'coercion'/exp<br>'intimidation'/exp<br>'dehumanization'/exp<br>'stalking'/de<br>'childhood adversity'/exp<br>'maltreatment'/exp<br>'corporal punishment'/exp<br>'victimization'/exp	'case control study'/exp<br>'crossover procedure'/exp<br>'cohort analysis'/exp<br>'systematic review'/exp<br>'systematic review (topic)'/exp<br>'meta analysis'/exp<br>'meta analysis (topic)'/exp<br>'twin study'/exp	'statistics'/exp<br>'statistical parameters'/exp<br>'risk'/exp<br>'risk ratio'/exp<br>'correlation'/exp<br>'statistical analysis'/exp
Free text terms (searched in title, abstract, and keyword (:ti,ab,kw))	violence<br>'sexual assault'<br>'sexual harassment'<br>'sexual abuse'<br>'sex abuse'<br>rape<br>'forced sex'<br>'sexual coercion'<br>'reproductive coercion'<br>'sex trafficking'<br>'sexual exploitation'<br>'forced marriage'	'systematic review'<br>meta-analysis<br>cohort<br>cross-over<br>case-control<br>prospective<br>retrospective<br>longitudinal<br>follow-up<br>followup	'risk*'<br>'odds'<br>'cross-product ratio*'<br>'hazards ratio*'<br>'hazard ratio*'<br>statistic*<br>'HR'<br>'RR'<br>'aOR'<br>relation*<br>correlat*<br>associat*

For peer review only

	'female genital mutilation'<br>'female genital cutting'<br>'female circumcision'<br>'female genital circumcision'<br>'physical abuse'<br>'psychological abuse'<br>'emotional abuse'<br>'economic abuse'<br>'verbal abuse'<br>maltreatment<br>'violent discipline'<br>'corporal punishment'<br>'adverse childhood experience*'<br>molestation<br>'child abuse'<br>'partner abuse'<br>'dating abuse'<br>'wife abuse'<br>'spouse abuse'<br>'domestic abuse'<br>'elder abuse'<br>'senior abuse'<br>'aged abuse'<br>victimization<br>dehumanization<br>victimisation<br>dehumanisation<br>stalking<br>cyberviolence<br>cybervictimization<br>cyberstalking		

For peer review only

Supplementary Table 2. Search Terms for CINAHL.

	**Concept: Violence**	**Concept: Study type**	**Concept: Risk**
Subject Headings (MH)	(MH "Sexual Abuse+")<br>(MH "Violence")<br>(MH "Domestic Violence+")<br>(MH "Gender-Based Violence")<br>(MH "Circumcision, Female")<br>(MH "Dating Violence")<br>(MH "Torture")<br>(MH "Workplace Violence")<br>(MH "Gun Violence")<br>(MH "Battered Women")<br>(MH "Child Abuse Survivors")<br>(MH "Exposure to Violence")<br>(MH "Emotional Abuse")<br>(MH "Sexual Harassment")<br>(MH "Aggression")<br>(MH "Verbal Abuse")<br>(MH "Coercion")<br>(MH "Dehumanization")<br>(MH "Stalking")<br>(MH "Adverse Childhood Experiences")<br>(MH "Sibling Violence")<br>(MH "School Violence")<br>(MH "Student Abuse")	(MH "Case Control Studies+")<br>(MH "Crossover Design")<br>(MH "Systematic Review")<br>(MH "Meta Analysis")<br>(MH "Prospective Studies+")<br>(MH "Retrospective Design")	(MH "Statistics+")<br>(MH "Data Analysis, Statistical+")
Free text terms (searched in Title & Abstract; keyword not a searchable field)	violence<br>"sexual assault"<br>"sexual harassment"<br>“sexual abuse”<br>“sex abuse”<br>rape<br>“forced sex”<br>“sexual coercion”<br>“reproductive coercion”<br>“sex trafficking”<br>“sexual exploitation”<br>“forced marriage”	"systematic review"<br>meta-analysis<br>cohort<br>cross-over<br>case-control<br>prospective<br>retrospective<br>longitudinal<br>follow-up<br>followup	risk*<br>odds<br>"cross-product ratio*"<br>"hazards ratio*"<br>"hazard ratio*"<br>statistic*<br>"HR"<br>"RR"<br>"aOR"<br>relation*<br>correlat*<br>associat*

For peer review only

	"female genital mutilation"<br>"female genital cutting"<br>"female circumcision"<br>"female genital circumcision"<br>"physical abuse"<br>"psychological abuse"<br>"emotional abuse"<br>"economic abuse"<br>"verbal abuse"<br>maltreatment<br>"violent discipline"<br>"corporal punishment"<br>"adverse childhood experience*"<br>molestation<br>"child abuse"<br>"partner abuse"<br>"dating abuse"<br>"wife abuse"<br>"spouse abuse"<br>"domestic abuse"<br>"elder abuse"<br>"senior abuse"<br>"aged abuse"<br>victimization<br>dehumanization<br>victimisation<br>dehumanisation<br>stalking<br>cyberviolence<br>cybervictimization<br>cyberstalking		

For peer review only

Supplementary Table 3. Search Terms for PsycInfo.

	Concept: Violence	Concept: Study type	Concept: Risk
PsycInfo Subject Headings (DE)	DE(<br>"Sexual Violence"<br>"Sex Offenses"<br>"Sexual Abuse"<br>"Sexual Coercion"<br>"Sex Trafficking"<br>"Violence"<br>"Domestic Violence"<br>"Child Abuse"<br>"Elder Abuse"<br>"Circumcision"<br>"Intimate Partner Violence"<br>"Dating Violence"<br>"Physical Abuse"<br>"Physical Discipline"<br>"Punishment"<br>"Rape"<br>"Acquaintance Rape"<br>"Torture"<br>"Workplace Violence"<br>"Gun Violence"<br>"Battered Females"<br>"Exposure to Violence"<br>"Emotional Abuse"<br>"Sexual Harassment"<br>"Aggressive Behavior"<br>"Verbal Abuse"<br>"Coercion"<br>"Stalking"<br>"Childhood Adversity"<br>"School Violence"<br>"Police Violence"<br>"Victimization"<br>)	DE(<br>"Cohort Analysis"<br>"Followup Studies"<br>"Longitudinal Studies"<br>"Retrospective Studies"<br>"Prospective Studies"<br>"Systematic Review"<br>"Meta Analysis"<br>)	DE(<br>"Statistics"<br>"Statistical Analysis"<br>"Risk Assessment"<br>"Statistical Correlation"<br>)
MeSH terms (MA)	MA(<br>"Sex Offenses"<br>"Domestic Violence"<br>"Gender-Based Violence"<br>"Intimate Partner Violence"<br>"Physical Abuse"<br>"Rape"	MA(<br>"Case-Control Studies"<br>"Cross-Over Studies"<br>"Cohort Studies"<br>)	MA(<br>"Statistics as Topic"<br>Risk<br>Odds Ratio<br>)

For peer review only

	"Torture"<br>"Workplace Violence"<br>"Gun violence"<br>"Battered Women"<br>"Adult Survivors of Child abuse"<br>"Exposure to Violence"<br>"Emotional Abuse"<br>"Sexual Harassment"<br>"Coercion"<br>"Dehumanization"<br>"stalking"<br>“adverse childhood experiences”<br>)		
Free text terms (searched in Title, Abstract, and Keywords)	violence<br>"sexual assault"<br>"sexual harassment"<br>“sexual abuse”<br>“sex abuse”<br>rape<br>“forced sex”<br>“sexual coercion”<br>“reproductive coercion”<br>“sex trafficking”<br>“sexual exploitation”<br>“forced marriage”<br>“female genital mutilation”<br>“female genital cutting”<br>“female circumcision”<br>“female genital circumcision”<br>“physical abuse”<br>“psychological abuse”<br>“emotional abuse”<br>“economic abuse”<br>"verbal abuse"<br>maltreatment<br>“violent discipline”<br>“corporal punishment”<br>“adverse childhood experience*”<br>molestation<br>“child abuse”<br>“partner abuse”<br>"dating abuse"	"systematic review"<br>meta-analysis<br>cohort<br>cross-over<br>case-control<br>prospective<br>retrospective<br>longitudinal<br>follow-up<br>followup	risk*<br>odds<br>"cross-product ratio*"<br>"hazards ratio*"<br>"hazard ratio*"<br>statistic*<br>"HR"<br>"RR"<br>"aOR"<br>relation*<br>correlat*<br>associat*

For peer review only

	"wife abuse"<br>"spouse abuse"<br>"domestic abuse"<br>"elder abuse"<br>"senior abuse"<br>"aged abuse"<br>victimization<br>dehumanization<br>victimisation<br>dehumanisation<br>stalking<br>cyberviolence<br>cybervictimization<br>cyberstalking		

For peer review only

Supplementary Table 4. Search Terms for Global Index Medicus.

	Concept: Violence	Concept: Study type	Concept: Risk
Subject Descriptors	I01.198.240.748*<br>Violence<br>I01.198.240.856.350*<br>I01.198.240.856.463<br>I01.198.240.856.575*<br>I01.198.240.856.688<br>I01.198.240.748.640<br>I01.198.240.856.825<br>I01.198.240.856.912<br>I01.198.240.856.519<br>M01.975.155<br>M01.135.500<br>I01.880.735.900.869<br>I01.880.735.305<br>SP9.020.800.010<br>"Harassment, Non-Sexual"<br>"Aggression"<br>I01.880.604.316<br>Dehumanization<br>F01.145.813.191*<br>I01.880.735.223.500<br>I01.880.735.035	E05.318.372.500.500*<br>E05.318.370.150<br>E05.318.372.500.750*<br>V03.850<br>L01.178.682.759.575<br>V03.600<br>E05.318.370.500*<br>V03.900	E05.318.740*<br>E05.318.740.600.800*<br>E05.318.740.600.600
Title, abstract, subject (tw:())	violence<br>"sexual assault"<br>"sexual harassment"<br>"sexual abuse"<br>"sex abuse"<br>rape<br>"forced sex"<br>"sexual coercion"<br>"reproductive coercion"<br>"sex trafficking"<br>"sexual exploitation"<br>"forced marriage"<br>"female genital mutilation"<br>"female genital cutting"<br>"female circumcision"<br>"female genital circumcision"<br>"physical abuse"<br>"psychological abuse"<br>"emotional abuse"<br>"economic abuse"<br>"verbal abuse"	"systematic review"<br>"meta-analysis"<br>cohort<br>"cross-over"<br>"case-control"<br>Prospective<br>retrospective<br>longitudinal<br>"follow-up"<br>followup	risk*<br>odds<br>"cross-product ratio"<br>"cross-product ratios"<br>"hazards ratio"<br>"hazards ratios"<br>"hazard ratio"<br>"hazard ratios"<br>statistic*<br>"HR"<br>"RR"<br>"aOR"<br>relation*<br>correlat*<br>associat*

For peer review only

	maltreatment<br>"violent discipline"<br>"corporal punishment"<br>"adverse childhood experience"<br>"adverse childhood experiences"<br>molestation<br>"child abuse"<br>"partner abuse"<br>"dating abuse"<br>"wife abuse"<br>"spouse abuse"<br>"domestic abuse"<br>"elder abuse"<br>"senior abuse"<br>"aged abuse"<br>victimization<br>dehumanization<br>victimisation<br>dehumanisation<br>stalking<br>cyberviolence<br>cybervictimization<br>cyberstalking		

For peer review only

Supplementary Table 5. Search Terms for Cochrane.

	Concept: Violence	Concept: Study type	Concept: Risk
Subject Headings [mh].	[mh "Sex Offenses"].<br>[mh ^"Violence"].<br>[mh "Domestic Violence"].<br>[mh "Gender-Based Violence"].<br>[mh "Intimate Partner Violence"].<br>[mh "Physical Abuse"].<br>[mh "Rape"].<br>[mh "Torture"].<br>[mh "Workplace Violence"].<br>[mh "Gun violence"].<br>[mh "Battered Women"].<br>[mh "Adult Survivors of Child abuse"].<br>[mh "Exposure to Violence"].<br>[mh "Emotional Abuse"].<br>[mh "Sexual Harassment"].<br>[mh ^"Harassment, Non-Sexual"].<br>[mh ^"Aggression"].<br>[mh "Coercion"].<br>[mh "Dehumanization"].<br>[mh "stalking"].<br>[mh “adverse childhood experiences”].	[mh "Case-Control Studies"].<br>[mh "Cross-Over Studies"].<br>[mh "Cohort Studies"].<br>[mh "Systematic Review"].<br>[mh "Meta-Analysis"].<br>[mh "Twin Study"].	[mh "Statistics as Topic"].<br>[mh Risk].<br>[mh "Odds Ratio"].
Free text terms :ti,ab,kw	violence<br>"sexual assault"<br>"sexual harassment"<br>“sexual abuse”<br>“sex abuse”<br>rape<br>“forced sex”<br>“sexual coercion”<br>“reproductive coercion”<br>“sex trafficking”<br>“sexual exploitation”<br>“forced marriage”<br>“female genital mutilation”<br>“female genital cutting”<br>“female circumcision”<br>“female genital circumcision”	"systematic review"<br>"meta-analysis"<br>"cohort"<br>"cross-over"<br>"case-control"<br>"prospective"<br>"retrospective"<br>"longitudinal"<br>"follow-up"<br>"followup"	"risk"<br>"odds"<br>"cross-product ratio"<br>"cross-product ratios"<br>"hazards ratio"<br>"hazards ratios"<br>"hazard ratio"<br>"hazard ratios"<br>statistic*<br>"HR"<br>"RR"<br>"aOR"<br>relation*<br>correlat*<br>associat*

For peer review only

	"physical abuse"<br>"psychological abuse"<br>"emotional abuse"<br>"economic abuse"<br>"verbal abuse"<br>maltreatment<br>"violent discipline"<br>"corporal punishment"<br>"adverse childhood experience"<br>"adverse childhood experiences"<br>molestation<br>"child abuse"<br>"partner abuse"<br>"dating abuse"<br>"wife abuse"<br>"spouse abuse"<br>"domestic abuse"<br>"elder abuse"<br>"senior abuse"<br>"aged abuse"<br>victimization<br>dehumanization<br>victimisation<br>dehumanisation<br>stalking<br>cyberviolence<br>cybervictimization<br>cyberstalking		

For peer review only

Supplementary Table 6. Search Terms for Web of Science.

	Concept: Violence	Concept: Study type	Concept: Risk
Topic (searches title, abstract, author keywords, Keywords Plus®)	violence<br>"sexual assault"<br>"sexual harassment"<br>"sexual abuse"<br>"sex abuse"<br>rape<br>"forced sex"<br>"sexual coercion"<br>"reproductive coercion"<br>"sex trafficking"<br>"sexual exploitation"<br>"forced marriage"<br>"female genital mutilation"<br>"female genital cutting"<br>"female circumcision"<br>"female genital circumcision"<br>"physical abuse"<br>"psychological abuse"<br>"emotional abuse"<br>"economic abuse"<br>"verbal abuse"<br>Maltreatment<br>torture<br>"violent discipline"<br>"corporal punishment"<br>"adverse childhood experience*"<br>molestation<br>"child abuse"<br>"partner abuse"<br>"dating abuse"<br>"wife abuse"<br>"battered wom*n"<br>"spouse abuse"<br>"domestic abuse"<br>"elder abuse"<br>"senior abuse"<br>"aged abuse"<br>victimization<br>dehumanization<br>victimisation	"systematic review"<br>"meta-analysis"<br>cohort<br>cross-over<br>case-control<br>prospective<br>retrospective<br>longitudinal<br>follow-up<br>followup	"risk*"<br>"odds"<br>"cross-product ratio*"<br>"hazards ratio*"<br>"hazard ratio*"<br>statistic*<br>"HR"<br>"RR"<br>"aOR"<br>relation*<br>correlat*<br>associat*

For peer review only

	dehumanisation<br>stalking<br>cyberviolence<br>cybervictimization<br>cyberstalking		

For peer review only

BMJ Open

Estimating the global health impact of gender-based violence and violence against children: a systematic review and meta-analysis protocol

Journal:	*BMJ Open*
Manuscript ID	bmjopen-2022-061248.R1
Article Type:	Protocol
Date Submitted by the Author:	19-May-2022
Complete List of Authors:	Spencer, Cory; Institute for Health Metrics and Evaluation<br>Baeza, María Jose; University of Miami School of Nursing and Health Studies<br>Chandan, Jaidev Kaur; University of Warwick Warwick Medical School<br>Debure, Alexandra; University of Miami School of Nursing and Health Studies<br>Herbert, Molly; Institute for Health Metrics and Evaluation<br>Jewell, Teresa; University of Washington Health Sciences Library<br>Khalil, Mariam; Institute for Health Metrics and Evaluation<br>Lim, Rachel Qian Hui; Queen Mary University of London Barts and The London School of Medicine and Dentistry<br>Minhas, Sonica; Queen Mary University of London Barts and The London School of Medicine and Dentistry<br>Chandan, Joht Singh; University of Birmingham Institute of Applied Health Research<br>Gakidou, Emmanuela; Institute for Health Metrics and Evaluation<br>Metheny, Nicholas; University of Miami School of Nursing and Health Studies
**Primary Subject Heading**:	Global health
Secondary Subject Heading:	Epidemiology, Public health
Keywords:	EPIDEMIOLOGY, PUBLIC HEALTH, STATISTICS & RESEARCH METHODS

SCHOLARONE™
Manuscripts

Estimating the global health impact of gender-based violence and violence against children: a systematic review and meta-analysis protocol

Cory N Spencer[1], María Jose Baeza[2], Jaidev Kaur Chandan[3], Alexandra Debure[2], Molly Herbert[1], Teresa Jewell[4], Mariam Khalil[1], Rachel Qian Hui Lim[5], Sonica Minhas[5], Joht Singh Chandan[6]*, Emmanuela Gakidou[1]*, Nicholas Metheny[2]*

*Joint senior author

Corresponding author:

Cory N Spencer
Institute for Health Metrics and Evaluation
University of Washington
Population Health Building/Hans Rosling Center
3980 15th Ave. NE, Seattle, WA 98195 USA
UW Campus Box #351615
781-249-0364
corycns@uw.edu

Affiliations:

[1]Institute for Health Metrics and Evaluation, University of Washington, Seattle, WA, USA
[2]School of Nursing and Health Studies, University of Miami, Coral Gables, FL, USA
[3]Warwick Medical School, University of Warwick, Coventry, UK
[4]Health Sciences Library, University of Washington, Seattle, WA, USA
[5]Barts and the London School of Medicine and Dentistry, Queen Mary's University of London, London, UK
[6]Institute of Applied Health Research, University of Birmingham, Birmingham, UK

Word Count: 3996

For peer review only

For peer review only - http://bmjopen.bmj.com/site/about/guidelines.xhtml

Abstract

Introduction: Exposure to gender-based violence (GBV) and violence in childhood (VAC) can result in substantial morbidity and mortality. Previous reviews of health outcomes associated with GBV and VAC have focused on limited definitions of exposure to violence (i.e., intimate partner violence) and often investigate associations only with predefined health outcomes. In this protocol, we describe a systematic review and meta-analysis for a comprehensive assessment of the impact of violence exposure on health outcomes and health-related risk factors across the life-course.

Methods and analysis: Electronic databases (PubMed, Embase, CINAHL, PsycInfo, Global Index Medicus, Cochrane, and Web of Science Core Collection) will be searched from 1st January 1970 to 30th September 2021 and searches updated to the current date prior to final preparation of results. Reviewers will first screen titles and abstracts, and eligible articles will then be full-text screened and accepted should they meet all inclusion criteria. Data will be extracted using a standardized form with fields to capture study characteristics and estimates of association between violence exposure and health outcomes. Individual study quality will be assessed via six risk of bias criteria. For exposure-outcome pairs with sufficient data, evidence will be synthesized via a Meta-regression---Bayesian, regularized, trimmed model and confidence in the cumulative evidence assessed via the burden of proof risk function. Where possible, variations in associations by subgroup, i.e. age, sex or gender, will be explored.

Ethics and dissemination: Formal ethical approval is not required. Findings from this review will be used to inform improved estimation of GBV and VAC within the Global Burden of Disease Study. The review has been undertaken in conjunction with the Lancet Commission on GBV and the Maltreatment of Young People with the aim of providing new data insights for a report on the global response to violence.

PROSPERO Registration Number CRD42022299831

Keywords: Epidemiology; public health; gender-based violence; violence against children

For peer review only - http://bmjopen.bmj.com/site/about/guidelines.xhtml

Article Summary

Strengths and limitations of this study

- This review is the first effort to systematically identify and assess all health-related impacts of multiple and overlapping forms of gender-based violence and violence against children.
- Data analysis plans include meta-regression and burden of proof frameworks to synthesize all available evidence and provide policy-direct interpretations of associations.
- Findings from the review will be incorporated in the Global Burden of Disease Study, the most comprehensive observational epidemiological study to date and a critical tool for researchers, advocates, and decision makers.
- Challenges remain in the comparability of definitions of exposure to gender-based violence and violence against children and availability of high quality data for under-studied forms of violence (i.e. cyberviolence, stalking, elder abuse).

For peer review only - http://bmjopen.bmj.com/site/about/guidelines.xhtml

Background

Gender-based violence (including but not limited to intimate partner violence (IPV), elder abuse, and violence against women) and violence against children (VAC) are global public health issues associated with a substantial burden of morbidity and mortality. It is well known that the immediate consequences of both VAC and gender-based violence in adulthood (GBV) include physical injuries and death [1]. However, the medium- and longer-term consequences are less well understood, but have shown to span a variety of physical, mental, sexual, and reproductive health issues [2,3]. Until recently, the fields of VAC and GBV were largely siloed, stunting our understanding of how different exposure to violence influence each other across the lifespan. To address these challenges, the Sexual Violence Research Initiative (SVRI), UNICEF Innocenti, and WHO have recently developed a framework of guiding principles encouraging interaction of research in the field of violence epidemiology [4]. As international advocacy and research organizations push for the integration of these fields, a more fulsome understanding of the health impacts of violence across the life-course is needed [5,6].

The Global Burden of Diseases (GBD), Injuries, and Risk Factors Study has quantified the global disease and disability burden of two violence-related risk factors, intimate partner violence (IPV) and childhood sexual abuse (CSA), within a comparative risk assessment framework since 2010 [7–10]. An advantage of the comparative risk assessment framework is the ability to compare the relative contribution to disease and disability among several health risk factors. Indeed, country- and age-specific findings from the GBD have shown IPV to account for more overall disability-adjusted life years (DALYs) in women ages 15-49 than more traditionally highlighted health risk factors such as smoking [11,12]. On a global scale, IPV was estimated to account for 6.44 million (95% uncertainty interval (UI), 3.55-9.87 million) global DALYs among this group in 2019 while, by comparison, smoking contributed to 4.52 million (95% UI 3.87-5.23 million) global DALYs in the same population in 2019 [4]. Much of the estimated health impact stems from GBD meta-analyses of scientific literature, which have found IPV exposure to be associated with a 54% increased risk of depression and 59% increased risk for HIV infection [8]. Likewise, those exposed to CSA have been estimated to be 2.21 times as likely to experience alcohol use disorder (relative risk (RR)=2.21, 95% UI=1.15-4.04) and 1.56 times as likely to experience depression (RR=1.56, 95% UI=1.30-1.86), accounting for 3.67 million (95% UI, 1.75-6.56 million) DALYs among males and females of all ages in 2019 [8]. While these findings provide a basis for understanding the impact of violence on health, the lack of a comprehensive analysis of the longitudinal literature has so far precluded the ability to expand the types of violence included in the GBD as well as the specific health outcomes that comprise the final estimates of burden. A more complete understanding of the adverse health outcomes associated with exposure to more types of GBV and VAC, and the magnitude of these associations, is needed to capture the negative health and societal impacts of GBV and VAC.

Beyond the estimates provided by the internationally comparative GBD studies, existing reviews assessing the health impacts of GBV and VAC have typically focused on the impact of a

For peer review only - http://bmjopen.bmj.com/site/about/guidelines.xhtml

single type of violence (e.g. IPV) on a specific health outcome (e.g. HIV). In 2013, the World Health Organization (WHO), London School of Hygiene and Tropical Medicine, and South African Medical Research Council conducted a systematic review and meta-analysis of a variety of health effects related to specific forms of GBV, measured as physical and/or sexual IPV or non-partner sexual violence (NPSV) [2]. Across studies identified, women exposed to IPV were 1.5 times as likely to become infected with HIV/AIDS and 1.97 times as likely to experience depression, among other adverse health outcomes [2]. A lack of comparable studies prevented meta-analysis for NPSV [1]. Following the publication of 2013 report, the WHO curated an extensive database of studies describing the literature explaining the relationship between violence against women (VAW) and VAC with subsequent health outcomes [13]. However, the database is not regularly maintained and has not updated summaries of high-quality evidence on the health impacts of GBV (VAW/IPV) since 2013, which is urgently needed to inform global health policy. In 2018, Bacchus et al. additionally reviewed cohort studies that reported on all health outcomes and behaviors related to recent physical and sexual IPV exposure, finding evidence of a positive, bidirectional relationship between these types of IPV and depressive symptoms [14]. However, there are fewer reviews investigating exposure types beyond physical and sexual IPV (e.g. psychological violence, coercive control, financial abuse, stalking), and those available often define even finer scopes by investigating relationships between narrowly defined forms of violence and narrowly defined health outcomes, including mental health and gynecological morbidity [15,16].

Similar to GBV, there have been attempts to synthesize the literature exploring the breadth of consequences following exposure to VAC and highlighting the relationship between exposure to childhood maltreatment (including CSA) and a wide variety of psychosocial and health outcomes [17,18]. Research into the consequences of VAC has more recently overlapped with the burgeoning literature base describing health outcomes secondary to adverse childhood experience (ACE) exposure, two of which include direct exposure to violence and the witnessing of parental IPV [19]. A recent comprehensive review highlighted the pervasive harms that ACEs place on health throughout the life course [20] and in a secondary analysis found that within Europe and North America, a 10% reduction in ACE prevalence could equate to annual savings of 3 million DALYs or $105 billion [21]. However, despite these efforts to capture the literature on VAC through either exploring childhood maltreatment, CSA, or ACEs as the marker of exposure, included studies are often limited to exposure in high income countries and exclude other forms of violence such as female genital mutilation, trafficking, forced marriage and cyberviolence.

While the existing literature has illuminated the significant health impacts of violence, critical evidence gaps remain. These include the quantification of the health burden of less-studied forms of violence, the health burden of violence in in lower- and middle-income settings, as well as the integration of violence in childhood and adulthood as an intergenerational issue that could be more effectively measured using a life-course approach. The life-course approach as outlined in the Minsk Declaration which essentially recognizes that all stages of a person's life

For peer review only - http://bmjopen.bmj.com/site/about/guidelines.xhtml

are intricately intertwined with each other, with the lives of other people in society, and with past and future generations of their families [22,23]. In order to do adopt this approach effectively when considering the health effects of GBV/VAC, we must consider that violence can occur at any stage in one's life (pre-conception to death) but also the impact of such event can be inter-generational and societal. Additionally, as highlighted through the reviews cited above, the current research trajectory often creates distinctions between GBV/VAC and other forms of life course violence such as elder-abuse [18]. However, considering that GBV and VAC share risk factors, co-occur and can lead to compounding consequences across the life course, there is a clear need to examine these phenomena in unison.

We present a systematic review and meta-analysis protocol to generate estimates of a comprehensive range of health impacts associated with exposure to GBV and VAC. Findings will contribute to the assessment of risk-outcome relationships and attributable burdens of disease within the GBD. To our knowledge, there has been no other systematic review and meta-analysis conducted with such a life-course approach across multiple types of violence.

Methods and analysis

The presentation of our review design follows the Preferred Reporting Items for Systematic review and Meta-Analysis Protocols (PRISMA-P) guidelines (Supplemental File 1) [24]. This review was registered in the PROSPERO International Prospective Register of systematic reviews on 1 February 2022.

Aims of the review

The aim of this review is to identify and synthesize all available data on the health impacts of exposure to any form of GBV and VAC. This data can then be used to assess risk-outcome relationships and quantify their contribution to global disease and disability burdens.

Specific review questions

1. How does exposure to GBV and/or VAC impact health across the life course?
2. What is the strength of evidence on the associations between exposure to GBV and VAC and different health impacts?
3. Do estimates of association vary by characteristics of the violence, global region, characteristics of the perpetrator, and/or characteristics of the victim?

Definitions

Definitions of violence

We include in our searches the following terms describing exposure to GBV and/or VAC:

- Gender-based violence;
- Intimate partner violence, partner abuse/violence, wife/spouse abuse, dating abuse/violence;
- Sexual abuse, rape, forced sex, sexual assault, sexual coercion, sexual exploitation;
- Reproductive coercion;

For peer review only - http://bmjopen.bmj.com/site/about/guidelines.xhtml

- Female genital mutilation, female genital cutting, female circumcision;
- Sex trafficking, child, early and forced marriage;
- Physical abuse;
- Psychological abuse, emotional abuse, verbal abuse;
- Economic abuse, financial abuse;
- Cyberviolence, cybervictimization;
- Domestic violence/abuse;
- Adverse childhood experiences that include direct exposure to and witnessing of violence;
- Child maltreatment, molestation, child abuse;
- Elder abuse, senior abuse, aged abuse;
- Stalking, cyberstalking;
- Dehumanization, torture;
- Workplace violence, student abuse, sexual harassment;
- Gender-based violence perpetrated with a firearm.

We expect author definitions and methods used to measure exposure to vary and will accept all definitions, documenting study definitions and measurement techniques as a part of study-level quality assessment.

Health outcomes

We did not restrict searches to pre-defined health outcomes and aim to accept all literature reporting an association between violence exposure and health. Definitions of health outcomes and health-related risk factors will be guided by causes, injuries, and risk factor case definitions from the Global Burden of Diseases study [8,25]. Studies that report on certain biomarkers or without accompanying clinical diagnoses (i.e., neural connectivity patterns, salivary cortisol as a stress response, DNA characteristics) will not be eligible for inclusion. Similarly, studies that report on the presence or number of disease symptoms without an accompany diagnosis of a health outcome will not be eligible for inclusion. Reviewers will meet regularly to raise questions about eligible health outcomes, with consensus decisions documented and circulated via written guidelines. Differences in measurement methods or case definitions of eligible health outcomes will be documented as a part of quality assessment as well. Final selection of associations to be synthesized will depend on the availability of studies that examine the association between a comparable form of exposure and reported health outcome.

Criteria for considering studies for this review

Inclusion

- Study design: Case-control, cohort, or case-crossover studies.
- Participants: Studies conducted in participant groups likely to be generalizable to the population of interest. Exposed groups are defined as any individual who has experienced a form of GBV and/or VAC throughout the lifetime. Comparators will be

For peer review only - http://bmjopen.bmj.com/site/about/guidelines.xhtml

non-exposed control groups, or study groups without reported exposure to a form of GBV and/or VAC.

- Outcomes: Studies reporting an estimate of association (either relative risk, risk ratio, odds-ratio, hazard ratio, or similar) or reporting cases and non-cases among those exposed and unexposed. If not provided directly, studies providing enough information to allow an estimate of relative to risk to be calculated will meet inclusion criteria.

Exclusion

- Study design: Cross-sectional, ecological, case series or case studies. We exclude cross-sectional studies in accordance with Global Burden of Disease study risk factor analyses, which typically do not include cross-sectional studies. This exclusion reason is related to the inability to assess temporality between exposures and outcomes in cross-sectional studies. We do not anticipate there to be any experimental studies, however, these will also be excluded.
- Participants: Studies conducted in subgroups identified only by convenience sampling or subgroups identified via a shared characteristic that is likely related to risk of exposure to violence or the reported health outcome (e.g. domestic violence shelter residents).
- Exposure measurement: Studies that report only an aggregate measure of exposure combining exposure to a form of violence with other, non-eligible exposures (e.g., reports a composite adverse childhood experience score only) will be excluded. For these studies, we are unable to disentangle the effect of violence exposure from the effects of other hardships or exposure types, preventing their inclusion in our review.
- Does not meet minimum reporting criteria: Studies missing essential data, i.e., do not report effect sizes and uncertainty information (confidence intervals, sample sizes) or the data needed to impute an effect size with uncertainty information.
- Studies reporting on the same exposure and outcome using the same data: The study with the longest follow-up time period or most complete dataset will be included.

Search strategy for identifying relevant studies

PubMed, Embase, CINAHL, PsycInfo, Global Index Medicus, Cochrane, and Web of Science Core Collection were searched using controlled vocabulary and keyword search terms. All relevant studies published between January 1st 1970 and September 30th 2021 will be considered, regardless of language of publication or study setting. Immediately prior to preparing final results from the review and meta-analysis, searches will be updated to the current month to retrieve for inclusion any further studies identified. The search strategy for PubMed is provided in Table 1. The search terms for Embase, CINAHL, PsycInfo, Global Index Medicus, Cochrane, and Web of Science are provided in the Supplementary Tables 1, 2, 3, 4, 5, and 6, respectively (Supplemental File 2).

For peer review only - http://bmjopen.bmj.com/site/about/guidelines.xhtml

Table 1. Search Terms and Strategy for PubMed.

Search Terms	Concept
1. "Sex Offenses"[mh].<br>2. "Violence"[mh:noexp].<br>3. "Domestic Violence"[mh].<br>4. "Gender-Based Violence"[mh].<br>5. "Intimate Partner Violence"[mh].<br>6. "Physical Abuse"[mh].<br>7. "Rape"[mh].<br>8. "Torture"[mh].<br>9. "Workplace Violence"[mh].<br>10. "Gun violence"[mh].<br>11. "Battered Women"[mh].<br>12. "Adult Survivors of Child abuse"[mh].<br>13. "Exposure to Violence"[mh].<br>14. "Emotional Abuse"[mh].<br>15. "Sexual Harassment"[mh].<br>16. "Harassment, Non-Sexual"[mh:noexp].<br>17. "Emotional abuse"[mh].<br>18. "Aggression"[mh:noexp].<br>19. "Dehumanization"[mh].<br>20. "stalking"[mh].<br>21. “adverse childhood experiences”[mh].<br>22. violence[tiab].<br>23. "sexual assault"[tiab].<br>24. "sexual harassment"[tiab].<br>25. “sexual abuse”[tiab].<br>26. “sex abuse”[tiab].<br>27. rape[tiab].<br>28. “forced sex”[tiab].<br>29. “sexual coercion”[tiab].<br>30. “reproductive coercion”[tiab].<br>31. “sex trafficking”[tiab].<br>32. “sexual exploitation”[tiab].<br>33. “forced marriage”[tiab].<br>34. “child marriage*”[tiab].<br>35. “early marriage*”[tiab].<br>36. “child bride*”[tiab].<br>37. CEFM[tiab].<br>38. infibulation*[tiab].<br>39. clitoridectom*[tiab].<br>40. clitorectom*[tiab].<br>41. “ritual female genital surger*”[tiab].<br>42. FGM[tiab].<br>43. “female genital mutilation”[tiab].<br>44. “female genital cutting”[tiab].<br>45. “female circumcision”[tiab].	Violence exposure

For peer review only - http://bmjopen.bmj.com/site/about/guidelines.xhtml

46. “female genital circumcision”[tiab].<br>47. “physical abuse”[tiab].<br>48. “psychological abuse”[tiab].<br>49. “emotional abuse”[tiab].<br>50. “economic abuse”[tiab].<br>51. “financial abuse”[tiab].<br>52. "verbal abuse"[tiab].<br>53. maltreatment[tiab].<br>54. “violent discipline”[tiab].<br>55. “corporal punishment”[tiab].<br>56. “adverse childhood experience*”[tiab].<br>57. molestation[tiab].<br>58. “child abuse”[tiab].<br>59. “partner abuse”[tiab].<br>60. "dating abuse"[tiab].<br>61. “wife abuse”[tiab].<br>62. “spouse abuse”[tiab].<br>63. “domestic abuse”[tiab].<br>64. “elder abuse”[tiab].<br>65. “senior abuse”[tiab].<br>66. “aged abuse”[tiab].<br>67. victimization[tiab].<br>68. dehumanization[tiab].<br>69. victimisation[tiab].<br>70. dehumanisation[tiab].<br>71. stalking[tiab].<br>72. cyberviolence[tiab].<br>73. cybervictimization[tiab].<br>74. cyberstalking[tiab].<br>75. Or/1-74	
76. Case-Control Studies[mh].<br>77. Cross-Over Studies[mh].<br>78. Cohort Studies[mh].<br>79. Systematic Review[pt].<br>80. Meta-Analysis[pt].<br>81. "Twin Study"[pt].<br>82. “systematic review”[tiab].<br>83. “meta-analysis”[tiab].<br>84. “cohort”[tiab].<br>85. “cross-over”[tiab].<br>86. “case-control”[tiab].<br>87. “prospective”[tiab].<br>88. “retrospective”[tiab].<br>89. “longitudinal”[tiab].<br>90. “follow-up”[tiab].<br>91. “followup”[tiab].<br>92. Or/76-91	Study type
93. "Statistics as Topic"[mh].<br>94. Risk[mh].<br>95. Odds Ratio[mh].<br>96. “risk*”[tiab].<br>97. “odds”[tiab].	Risk

For peer review only

For peer review only - http://bmjopen.bmj.com/site/about/guidelines.xhtml

98. "cross-product ratio*"[tiab].<br>99. "hazards ratio*"[tiab].<br>100."hazard ratio*"[tiab].<br>101.statistic*[tiab].<br>102."HR"[tiab].<br>103."RR"[tiab].<br>104."aOR"[tiab].<br>105.relation*[tiab].<br>106.correlat*[tiab].<br>107.associat*[tiab].<br>108.likel*[tiab].<br>109.Or/93-108	
110."1970/01/01"[PDat]. : "2021/09/30"[PDat].	Date restriction – all available literature since 1970
111.75 AND 92 AND 109 AND 110	

Data management and extraction

Search results will be merged and duplicates removed using the systematic review management software Covidence [26]. All reviewers will initially screen the first 50 search results and meet to compare screening decisions and clarify any questions with regard to the inclusion criteria. The first two-thirds of titles and abstracts will be screened by two independent reviewers, and JSC, NM and CS will review and resolve all conflicts that arise during screening decisions. Upon confirmation of a low rate of conflicts (less than 10%) in the first two thirds of double-screened articles, the remaining third of articles will be screened by a single reviewer. This approach balances the priorities of independent review and completing our review in a timely manner. Non-English publications will be reviewed using the language fluencies (Spanish, French, and Portuguese) of the reviewers. Should articles in other languages be retrieved and eligible for extraction, the reviewers will contact colleagues fluent in these languages for assistance.

Reviewers will complete title and abstract screening of all articles before the team proceeds to full-text screening. Studies that met inclusion criteria in title and abstract screening will additionally be full-text screened and excluded if found to meet any of the exclusion criteria. Following the PRISMA guideline, each exclusion will include documentation of a specific exclusion reason. Within Covidence, there are several built-in exclusion reasons (i.e., wrong study design, wrong setting, etc.) as well as the possibility to create custom exclusion reasons. Reviewers will meet to discuss the addition of custom exclusion reasons prior to beginning full-text review, and will iteratively meet to discuss addition of new reasons as necessary. For full text review, 10% of articles will be reviewed by two independent reviewers and a meeting held to resolve misunderstandings and ensure all reviewers clearly understand inclusion and exclusion criteria. The remaining 90% of articles will be reviewed by one independent reviewer. If reviewers are unable to access the full text of a publication, the reviewers will reach out

For peer review only - http://bmjopen.bmj.com/site/about/guidelines.xhtml

directly to the corresponding author and wait a maximum of one month for response, after which point the article will be excluded.

Data extraction will occur in parallel with full-text review, with some team members beginning to extract data once a sufficient number of full-text articles have been accepted. Before any reviewer begins data extraction, the entire review team will conduct a consensus building exercise in which all reviewers extract data from the same 10 accepted articles. In a group meeting, extractions will be compared and any questions resolved. Reviewers will extract data from accepted articles using a standardized form created in Covidence [26]. The data extraction form will include variables related to (1) characteristics of the study, (2) characteristics of the study population, (3) exposure and outcome measurement, (4) effect size and uncertainty, (5) risk of bias (quality assessment) [27,28]. Data items are provided in Table 2.

Table 2. Variables to be collected in the data extraction process.

Category	Data items
1. Study characteristics	- author(s)<br>- citation<br>- year of publication<br>- geographical location<br>- study year(s)<br>- study design<br>- study name
2. Population characteristics	- selection criteria<br>- age summary statistics (range or median and standard deviation)<br>- sex or gender summary statistics (percent women or percent female)<br>- follow-up duration
3. Exposure and outcome measurement	- exposure definition<br>- exposure assessment frequency<br>- exposure recall period<br>- exposure type included in relative risk estimation<br>- exposure categories

For peer review only - http://bmjopen.bmj.com/site/about/guidelines.xhtml

	- outcome definition
4. Effect size and uncertainty	- effect size measure (e.g., relative risk, odds ratio, incidence rate ratio, hazard ratio)<br>- effect size<br>- confidence interval and level<br>- non-confidence interval uncertainty type and value<br>- sample size (total, exposed, unexposed)<br>- person-time (total, exposed, unexposed)<br>- number of events (total, among exposed, among unexposed)<br>- whether main or subgroup analysis<br>- description of subgroup analysis
5. Risk of bias (quality assessment)	- exposure assessment method<br>- exposure assessment instrument<br>- outcome assessment method<br>- outcome assessment instrument<br>- representativeness<br>- confounders adjusted for in reported effect size<br>- percent of participants for which exposure data was obtained (for case-control studies)<br>- drop-out rate (for cohort studies)<br>- risk of reverse causation

If a study reports on multiple forms of violence exposure, multiple associated health outcomes, or reports findings by subgroup or model specification, data pertaining to each sub-analysis will be extracted in addition to any aggregate results. In the case of a study reporting effect sizes for multiple model specifications, the most appropriately adjusted model will be selected for inclusion in meta-analysis.

Risk of bias in individual studies

Sources of bias will be assessed and collected during data extraction. Following the Grading of Recommendations, Assessment, Development and Evaluations (GRADE) approach [28], risk of bias criteria for individual studies include,

For peer review only - http://bmjopen.bmj.com/site/about/guidelines.xhtml

- Exposure measurement: How exposure to violence was assessed (whether standard, acts-based and specific questions were asked, e.g. "Have you ever been shoved, slapped, hit, or kicked by an intimate partner?" versus questions that rely upon participants' own definition of abuse, e.g. "Have you ever experienced domestic abuse?"). In addition, whether exposure was based upon self-reports or another source (e.g. health records).
- Outcome measurement: How reported health outcome(s) were measured (by physician diagnosis, diagnostic survey instruments, electronic health records, or reported as symptom severity or symptom scales).
- Representativeness of study population: If a study sample was based on the general population or if study results are reported from a sub-group for which there are prior reasons to believe that findings would be different.
- Control for confounding: If a study statistically controlled for confounding using all major known confounders, including age, sex, education, income, and other critical determinants of the health outcome.
- Selection bias: If a study is at risk of selection bias, based upon percent follow-up for longitudinal study designs and based upon the percentages of cases and controls for which exposure data can be ascertained for case-control designs.
- Reverse causation: If a study is at risk of reverse causation, evaluated through study design and opportunity for recall bias (i.e. case-control studies) [29].

Data Synthesis

If there are at least 3 studies identified with a comparable form of exposure and reported health outcome, we will synthesize effect sizes using a Meta-regression---Bayesian, regularized, trimmed (MR-BRT) model [8,30].

For each risk-outcome pair identified, we will use the MR-BRT tool to perform a meta-regression analysis of the risk of the given outcome for those exposed to the violence type relative to the reference category of those not exposed to the violence type. For risk-outcome pairs with sufficient data points, we will introduce likelihood-based trimming to detect and remove outliers before fitting the model, with an inlier fraction of 90% [30].

For each risk-outcome pair meta-analysis, we will consider study-level covariates with the potential to bias the study's reported effect size estimates and adjust for these covariates if they are found to significantly bias the estimated relative risk. The MR-BRT tool includes an automated covariate selection process using a Lasso strategy to identify statistically significant covariates at a significance threshold of 0.05 [30,31].

The MR-BRT tool quantifies between-study heterogeneity by accounting for heterogeneity uncertainty and small numbers of studies [30]. In this approach, the Fisher information matrix is used to estimate uncertainty of the between-study heterogeneity parameter, γ [30,32]. The final uncertainty estimate reflects both the posterior uncertainty corresponding to the fixed

For peer review only - http://bmjopen.bmj.com/site/about/guidelines.xhtml

effect and the 95% quantile of γ, which is sensitive to the number of studies, study design, and reported uncertainty of the effect size [30].

For each risk-outcome pair, we will additionally test for and report publication bias in the input data based upon the Egger's Regression strategy, which tests the degree to which standard error is correlated with effect size in the data, and present funnel plots [33,34].

Additional Analyses

If meta-analysis is not possible with all studies, we will synthesize the included study findings graphically [35] following the systematic review without meta-analysis [SWiM] guidance. This will include forest plots, which will graphically depict all study effect estimates using a single metric (e.g., percent change) for each available health outcome and type of violence [36]. To produce the forest plots, we will transform effect estimates to a comparable metric wherever possible (i.e., where the necessary data are available in the paper or from the authors). Harvest plots will demonstrate where inequities based on, for example, age of exposure, low- and middle-income country vs. high-income country, gender identity, ethnicity/race, sexual orientation, urban-rural location, exist in the available data [37]. When the necessary data are missing, all study effect estimates will be summarized in supplementary tables and discussed as relevant in text. If the necessary data are available (standardized effect estimate, p-value) we will also consider albatross plots to summarize results [38].

Confidence in cumulative evidence

Confidence in risk-outcome pair results will be assessed via the burden of proof risk function (BPRF). For binary risk-outcome pairs, the BPRF is defined as the 5th quantile for the effect size (that includes the effect size uncertainty and the 95% quantile of between-study heterogeneity) closest to the null (log relative risk of 0) and can be interpreted as the smallest effect consistent with available evidence [39]. A risk-outcome score (ROS) and corresponding star-rating will be derived from the BPRF. Star-rating categories are a policy-direct way to interpret the evidence for risk-outcome pairs, with higher star-ratings indicating stronger evidence of an association [39].

Narrative Synthesis

Narrative synthesis will be conducted by grouping studies according to exposure type and health outcomes. We will explore the breadth of available evidence across groupings as well as highlight the health outcomes and violence types for which there is stronger evidence than others, drawing upon results from meta-analyses and risk-outcome scores. The description of these patterns will allow us to make recommendations for future research as well as discuss the ways in which distinct types of violence affect health.

Patient and public involvement

Patients and/or the public were not involved in the design, or conduct, or reporting, or dissemination plans of this research.

For peer review only - http://bmjopen.bmj.com/site/about/guidelines.xhtml

Ethics and Dissemination

The proposed review does not require formal ethical approval. Findings from this review will contribute to GBD estimates of the health impact of GBV and VAC. The GBD includes data on morbidity and mortality from 1990 to present in 204 countries and territories for 369 diseases and injuries and 87 risk factors [40]. It is the most comprehensive worldwide observational epidemiological study to date and a critical tool used by clinicians, policy makers, and researchers. Review findings will inform the GBD assessment of new risks and/or risk-outcome relationships and revisions to the magnitude of currently included associations. Updated global health estimates of the impact of GBV and VAC will be highlighted in consequent GBD releases and accompanying capstone publications.

In addition, this review is being conducted in conjunction with the Lancet Commission on Gender-Based Violence and the Maltreatment of Young People [41]. The aim of the Commission is to complete a path breaking report on the global response to violence across the life course, complete with new data insights and concrete policy and research recommendations that are informed by survivors and advocates. This report will be published by The Lancet in an effort to initiate debate, offer insight and explanation, and influence decision makers across the globe regarding GBV and VAC.

Author Contributions

The initial PubMed search strategy was developed by CS and refined and adapted to other databases by TJ. The writing and methodological plan for this protocol was developed by CS, JSC, NM, and EG. Further revisions to the protocol were made by all remaining authors (MB, AD, MH, TJ, JKC, MK, RL, and SM) and the final copy of the manuscript was approved by all authors.

Funding statement

This work was supported by the Bill and Melinda Gates foundation grant number INV-018617. The funders of had no role in the review design or the writing of the report.

Competing interests statement

None declared.

Data Availability Statement

No data are available from this protocol.

References

1 Stöckl H, Devries K, Rotstein A, *et al.* The global prevalence of intimate partner homicide: a systematic review. *Lancet Lond Engl* 2013;**382**:859–65. doi:10.1016/S0140-6736(13)61030-2

For peer review only

For peer review only - http://bmjopen.bmj.com/site/about/guidelines.xhtml

2 WHO, Department of Reproductive Health and Research, London School of Hygiene and Tropical Medicine, South African Medical Research Council. Global and regional estimates of violence against women: prevalence and health effects of intimate partner violence and non-partner sexual violence.

3 The Lancet. Adversity in childhood: how the past defines the future. *Lancet Lond Engl* 2016;**388**:2324. doi:10.1016/S0140-6736(16)32172-9

4 UNICEF Office of Research-Innocenti. Joining forces to develop a research agenda on intersections of violence against children and violence against women. UNICEF-IRC. https://www.unicef-irc.org/article/2121-joining-forces-to-develop-a-research-agenda-on-intersections-of-violence-against-children-and-violence.html (accessed 29 Nov 2021).

5 Understanding the Linkages Between Violence Against Women and Violence Against Children. Spotlight Initiative https://endvawnow.org/uploads/browser/files/spotlight_initiative_guidance_on_linkages_between_vaw+vac.pdf

6 Intersections of Violence Against Women and Violence Against Children. Prev. Collab. 2021.https://prevention-collaborative.org/about-violence/intersections-of-vaw-and-vac/, https://prevention-collaborative.org/about-violence/intersections-of-vaw-and-vac/ (accessed 16 May 2022).

7 Lim SS, Vos T, Flaxman AD, *et al.* A comparative risk assessment of burden of disease and injury attributable to 67 risk factors and risk factor clusters in 21 regions, 1990-2010: a systematic analysis for the Global Burden of Disease Study 2010. *Lancet Lond Engl* 2012;**380**:2224–60. doi:10.1016/S0140-6736(12)61766-8

8 GBD 2019 Risk Factors Collaborators. Global burden of 87 risk factors in 204 countries and territories, 1990-2019: a systematic analysis for the Global Burden of Disease Study 2019. *Lancet Lond Engl* 2020;**396**:1223–49. doi:10.1016/S0140-6736(20)30752-2

9 Murray CJ, Lopez AD. Global mortality, disability, and the contribution of risk factors: Global Burden of Disease Study. *Lancet Lond Engl* 1997;**349**:1436–42. doi:10.1016/S0140-6736(96)07495-8

10 Murray CJ, Ezzati M, Lopez AD, *et al.* Comparative quantification of health risks conceptual framework and methodological issues. *Popul Health Metr* 2003;**1**:1. doi:10.1186/1478-7954-1-1

11 Vos T, Astbury J, Piers LS, *et al.* Measuring the impact of intimate partner violence on the health of women in Victoria, Australia. *Bull World Health Organ* 2006;**84**:739–44.

12 Mokdad AH, Forouzanfar MH, Daoud F, *et al.* Global burden of diseases, injuries, and risk factors for young people's health during 1990–2013: a systematic analysis for the Global

For peer review only - http://bmjopen.bmj.com/site/about/guidelines.xhtml

Burden of Disease Study 2013. *The Lancet* 2016;**387**:2383–401. doi:10.1016/S0140-6736(16)00648-6

13 World Health Organization. Violence Info – A global knowledge platform for preventing violence. http://apps.who.int/violence-info/ (accessed 29 Nov 2021).

14 Bacchus LJ, Ranganathan M, Watts C, *et al.* Recent intimate partner violence against women and health: a systematic review and meta-analysis of cohort studies. *BMJ Open* 2018;**8**:e019995. doi:10.1136/bmjopen-2017-019995

15 Hassam T, Kelso E, Chowdary P, *et al.* Sexual assault as a risk factor for gynaecological morbidity: An exploratory systematic review and meta-analysis. *Eur J Obstet Gynecol Reprod Biol* 2020;**255**:222–30. doi:10.1016/j.ejogrb.2020.10.038

16 Devries KM, Mak JY, Bacchus LJ, *et al.* Intimate partner violence and incident depressive symptoms and suicide attempts: a systematic review of longitudinal studies. *PLoS Med* 2013;**10**:e1001439. doi:10.1371/journal.pmed.1001439

17 Gilbert R, Widom CS, Browne K, *et al.* Burden and consequences of child maltreatment in high-income countries. *Lancet Lond Engl* 2009;**373**:68–81. doi:10.1016/S0140-6736(08)61706-7

18 Hailes HP, Yu R, Danese A, *et al.* Long-term outcomes of childhood sexual abuse: an umbrella review. *Lancet Psychiatry* 2019;**6**:830–9. doi:10.1016/S2215-0366(19)30286-X

19 Centers for Disease Control and Prevention. Adverse Childhood Experiences (ACEs). 2021.https://www.cdc.gov/violenceprevention/aces/index.html (accessed 9 Dec 2021).

20 Hughes K, Bellis MA, Hardcastle KA, *et al.* The effect of multiple adverse childhood experiences on health: a systematic review and meta-analysis. *Lancet Public Health* 2017;**2**:e356–66. doi:10.1016/S2468-2667(17)30118-4

21 Bellis MA, Hughes K, Ford K, *et al.* Life course health consequences and associated annual costs of adverse childhood experiences across Europe and North America: a systematic review and meta-analysis. *Lancet Public Health* 2019;**4**:e517–28. doi:10.1016/S2468-2667(19)30145-8

22 The Minsk Declaration: The Life-course Approach in the Context of Health 2020. World Health Organization Regional Office for Europe https://www.euro.who.int/__data/assets/pdf_file/0009/289962/The-Minsk-Declaration-EN-rev1.pdf

23 The life-course approach: from theory to practice. Case stories from two small countries in Europe. World Health Organization Regional Office for Europe https://www.euro.who.int/__data/assets/pdf_file/0004/374359/life-course-iceland-malta-eng.pdf

For peer review only

For peer review only - http://bmjopen.bmj.com/site/about/guidelines.xhtml

24 Moher D, Shamseer L, Clarke M, *et al.* Preferred reporting items for systematic review and meta-analysis protocols (PRISMA-P) 2015 statement. *Syst Rev* 2015;**4**:1. doi:10.1186/2046-4053-4-1

25 Vos T, Lim SS, Abbafati C, *et al.* Global burden of 369 diseases and injuries in 204 countries and territories, 1990–2019: a systematic analysis for the Global Burden of Disease Study 2019. *The Lancet* 2020;**396**:1204–22. doi:10.1016/S0140-6736(20)30925-9

26 *Covidence systematic review software*. Melbourne, Australia: : Veritas Health Innovation www.covidence.org (accessed 3 Nov 2021).

27 Wells G, Shea B, O'Connel D, *et al.* The Newcastle-Ottawa Scale (NOS) for assessing the quality of nonrandomised studies in meta-analyses.Ottawa Hospital Research Institute. Published Online First: 2013.http://www.ohri.ca/programs/clinical_epidemiology/oxford.asp, (accessed 29 Nov 2021).

28 Guyatt GH, Oxman AD, Vist G, *et al.* GRADE guidelines: 4. Rating the quality of evidence--study limitations (risk of bias). *J Clin Epidemiol* 2011;**64**:407–15. doi:10.1016/j.jclinepi.2010.07.017

29 Chouinard E, Walter S. Recall bias in case-control studies: an empirical analysis and theoretical framework. *J Clin Epidemiol* 1995;**48**:245–54. doi:10.1016/0895-4356(94)00132-a

30 Zheng P, Barber R, Sorensen RJD, *et al.* Trimmed Constrained Mixed Effects Models: Formulations and Algorithms. *J Comput Graph Stat* 2021;**30**:544–56. doi:10.1080/10618600.2020.1868303

31 Tibshirani R. Regression Shrinkage and Selection via the Lasso. *J R Stat Soc Ser B Methodol* 1996;**58**:267–88.

32 Casella G, Berger RL. *Statistical Inference*. Cengage Learning 2021.

33 Egger M, Smith GD, Schneider M, *et al.* Bias in meta-analysis detected by a simple, graphical test. *BMJ* 1997;**315**:629–34. doi:10.1136/bmj.315.7109.629

34 Sterne JAC, Egger M. Regression Methods to Detect Publication and Other Bias in Meta-Analysis. In: *Publication Bias in Meta-Analysis*. John Wiley & Sons, Ltd 2005. 99–110. doi:10.1002/0470870168.ch6

35 Campbell M, McKenzie JE, Sowden A, *et al.* Synthesis without meta-analysis (SWiM) in systematic reviews: reporting guideline. *BMJ* 2020;**368**:l6890. doi:10.1136/bmj.l6890

For peer review only - http://bmjopen.bmj.com/site/about/guidelines.xhtml

36 Humphreys DK, Ogilvie D. Synthesising evidence for equity impacts of population-based physical activity interventions: a pilot study. *Int J Behav Nutr Phys Act* 2013;**10**:76. doi:10.1186/1479-5868-10-76

37 Ogilvie D, Fayter D, Petticrew M, *et al.* The harvest plot: A method for synthesising evidence about the differential effects of interventions. *BMC Med Res Methodol* 2008;**8**:8. doi:10.1186/1471-2288-8-8

38 Harrison S, Jones HE, Martin RM, *et al.* The albatross plot: A novel graphical tool for presenting results of diversely reported studies in a systematic review. *Res Synth Methods* 2017;**8**:281–9. doi:10.1002/jrsm.1239

39 GBD 2020 Risk Score Collaborators. Burden of proof risk functions: a meta-analytic approach to assessing evidence. *Nat Med in review*

40 About the Global Burden of Disease. https://www.thelancet.com/gbd/about (accessed 29 Nov 2021).

41 Knaul FM, Bustreo F, Horton R. Countering the pandemic of gender-based violence and maltreatment of young people: The Lancet Commission. *The Lancet* 2020;**395**:98–9. doi:10.1016/S0140-6736(19)33136-8

For peer review only

Reporting checklist for protocol of a systematic review and meta analysis.

Based on the PRISMA-P guidelines.

Instructions to authors

Complete this checklist by entering the page numbers from your manuscript where readers will find each of the items listed below.

Your article may not currently address all the items on the checklist. Please modify your text to include the missing information. If you are certain that an item does not apply, please write "n/a" and provide a short explanation.

Upload your completed checklist as an extra file when you submit to a journal.

In your methods section, say that you used the PRISMA-Preporting guidelines, and cite them as:

Moher D, Shamseer L, Clarke M, Ghersi D, Liberati A, Petticrew M, Shekelle P, Stewart LA. Preferred Reporting Items for Systematic Review and Meta-Analysis Protocols (PRISMA-P) 2015 statement. Syst Rev. 2015;4(1):1.

		Reporting Item	Page Number
**Title**			
Identification	#1a	Identify the report as a protocol of a systematic review	1
Update	#1b	If the protocol is for an update of a previous systematic review, identify as such	n/a
**Registration**			
	#2	If registered, provide the name of the registry (such as PROSPERO) and registration number	2
**Authors**			
Contact	#3a	Provide name, institutional affiliation, e-mail address of all protocol authors; provide physical mailing address of corresponding author	1

For peer review only

Contribution	#3b	Describe contributions of protocol authors and identify the guarantor of the review	16
**Amendments**			
	#4	If the protocol represents an amendment of a previously completed or published protocol, identify as such and list changes; otherwise, state plan for documenting important protocol amendments	n/a. This protocol is not an amendment.
**Support**			
Sources	#5a	Indicate sources of financial or other support for the review	16
Sponsor	#5b	Provide name for the review funder and / or sponsor	16
Role of sponsor or funder	#5c	Describe roles of funder(s), sponsor(s), and / or institution(s), if any, in developing the protocol	16
**Introduction**			
Rationale	#6	Describe the rationale for the review in the context of what is already known	4-6
Objectives	#7	Provide an explicit statement of the question(s) the review will address with reference to participants, interventions, comparators, and outcomes (PICO)	6
**Methods**			
Eligibility criteria	#8	Specify the study characteristics (such as PICO, study design, setting, time frame) and report characteristics (such as years considered, language, publication status) to be used as criteria for eligibility for the review	7-8
Information sources	#9	Describe all intended information sources (such as electronic databases, contact with study authors, trial registers or other grey literature sources) with planned dates of coverage	8
Search strategy	#10	Present draft of search strategy to be used for at least one electronic database, including planned limits, such that it could be repeated	8-11; S2

For peer review only

Study records - data management	#11a	Describe the mechanism(s) that will be used to manage records and data throughout the review	11-12
Study records - selection process	#11b	State the process that will be used for selecting studies (such as two independent reviewers) through each phase of the review (that is, screening, eligibility and inclusion in meta-analysis)	12
Study records - data collection process	#11c	Describe planned method of extracting data from reports (such as piloting forms, done independently, in duplicate), any processes for obtaining and confirming data from investigators	11-12
Data items	#12	List and define all variables for which data will be sought (such as PICO items, funding sources), any pre-planned data assumptions and simplifications	12-14
Outcomes and prioritization	#13	List and define all outcomes for which data will be sought, including prioritization of main and additional outcomes, with rationale	7
Risk of bias in individual studies	#14	Describe anticipated methods for assessing risk of bias of individual studies, including whether this will be done at the outcome or study level, or both; state how this information will be used in data synthesis	13-14
Data synthesis	#15a	Describe criteria under which study data will be quantitatively synthesised	14-15
Data synthesis	#15b	If data are appropriate for quantitative synthesis, describe planned summary measures, methods of handling data and methods of combining data from studies, including any planned exploration of consistency (such as I2, Kendall's τ)	14-15
Data synthesis	#15c	Describe any proposed additional analyses (such as sensitivity or subgroup analyses, meta-regression)	15
Data synthesis	#15d	If quantitative synthesis is not appropriate, describe the type of summary planned	15
Meta-bias(es)	#16	Specify any planned assessment of meta-bias(es) (such as publication bias across studies, selective reporting within studies)	14-15

For peer review only

Confidence in cumulative evidence	#17	Describe how the strength of the body of evidence will be assessed (such as GRADE)	15

Notes:

- 4: n/a. This protocol is not an amendment.

The PRISMA-P elaboration and explanation paper is distributed under the terms of the Creative Commons Attribution License CC-BY. This checklist was completed on 13. January 2022 using https://www.goodreports.org/, a tool made by the EQUATOR Network in collaboration with Penelope.ai

For peer review only

Supplementary Table 1. Search Terms for Embase.

	Concept: Violence	Concept: Study type	Concept: Risk
Subject Headings (Emtree)	'sexual violence'/exp<br>'forced sex'/exp<br>'violence'/de<br>'domestic violence'/exp<br>'gender based violence'/exp<br>'partner violence'/exp<br>'dating violence'/exp<br>'physical abuse'/exp<br>'physical violence'/exp<br>'torture'/exp<br>'workplace violence'/exp<br>'gun violence'/exp<br>'battered woman'/exp<br>'child abuse survivor'/exp<br>'exposure to violence'/exp<br>'emotional abuse'/exp<br>'elderly abuse'/exp<br>'sexual harassment'/exp<br>'non-sexual harassment'/de<br>'aggression'/de<br>'verbal hostility'/exp<br>'coercion'/exp<br>'intimidation'/exp<br>'dehumanization'/exp<br>'stalking'/de<br>'childhood adversity'/exp<br>'maltreatment'/exp<br>'corporal punishment'/exp<br>'victimization'/exp	'case control study'/exp<br>'crossover procedure'/exp<br>'cohort analysis'/exp<br>'systematic review'/exp<br>'systematic review (topic)'/exp<br>'meta analysis'/exp<br>'meta analysis (topic)'/exp<br>'twin study'/exp	'statistics'/exp<br>'statistical parameters'/exp<br>'risk'/exp<br>'risk ratio'/exp<br>'correlation'/exp<br>'statistical analysis'/exp
Free text terms (searched in title, abstract, and keyword (:ti,ab,kw))	violence<br>'sexual assault'<br>'sexual harassment'<br>'sexual abuse'<br>'sex abuse'<br>rape<br>'forced sex'<br>'sexual coercion'<br>'reproductive coercion'<br>'sex trafficking'<br>'sexual exploitation'<br>'forced marriage*'	'systematic review'<br>meta-analysis<br>cohort<br>cross-over<br>case-control<br>prospective<br>retrospective<br>longitudinal<br>follow-up<br>followup	'risk*'<br>'odds'<br>'cross-product ratio*'<br>'hazards ratio*'<br>'hazard ratio*'<br>statistic*<br>'HR'<br>'RR'<br>'aOR'<br>relation*<br>correlat*<br>associat*

For peer review only

	'child marriage*'<br>'early marriage*'<br>'child bride*'<br>CEFM<br>'female genital mutilation'<br>'female genital cutting'<br>'female circumcision'<br>'female genital circumcision'<br>infibulation*<br>clitoridectom*<br>clitorectom*<br>'ritual female genital surger*'<br>FGM<br>'physical abuse'<br>'psychological abuse'<br>'emotional abuse'<br>'economic abuse'<br>'financial abuse'<br>'verbal abuse'<br>maltreatment<br>'violent discipline'<br>'corporal punishment'<br>'adverse childhood experience*'<br>molestation<br>'child abuse'<br>'partner abuse'<br>'dating abuse'<br>'wife abuse'<br>'spouse abuse'<br>'domestic abuse'<br>'elder abuse'<br>'senior abuse'<br>'aged abuse'<br>victimization<br>dehumanization<br>victimisation<br>dehumanisation<br>stalking<br>cyberviolence<br>cybervictimization<br>cyberstalking		likel*

Supplementary Table 2. Search Terms for CINAHL.

	Concept: Violence	Concept: Study type	Concept: Risk
Subject Headings (MH)	(MH "Sexual Abuse+")<br>(MH "Violence")<br>(MH "Domestic Violence+")<br>(MH "Gender-Based Violence")<br>(MH "Circumcision, Female")<br>(MH "Dating Violence")<br>(MH "Torture")<br>(MH "Workplace Violence")<br>(MH "Gun Violence")<br>(MH "Battered Women")<br>(MH "Child Abuse Survivors")<br>(MH "Exposure to Violence")<br>(MH "Emotional Abuse")<br>(MH "Sexual Harassment")<br>(MH "Aggression")<br>(MH "Verbal Abuse")<br>(MH "Coercion")<br>(MH "Dehumanization")<br>(MH "Stalking")<br>(MH "Adverse Childhood Experiences")<br>(MH "Sibling Violence")<br>(MH "School Violence")<br>(MH "Student Abuse")	(MH "Case Control Studies+")<br>(MH "Crossover Design")<br>(MH "Systematic Review")<br>(MH "Meta Analysis")<br>(MH "Prospective Studies+")<br>(MH "Retrospective Design")	(MH "Statistics+")<br>(MH "Data Analysis, Statistical+")
Free text terms (searched in Title & Abstract; keyword not a searchable field)	violence<br>"sexual assault"<br>"sexual harassment"<br>"sexual abuse"<br>"sex abuse"<br>rape<br>"forced sex"<br>"sexual coercion"<br>"reproductive coercion"<br>"sex trafficking"<br>"sexual exploitation"<br>"forced marriage*"<br>"child marriage*"	"systematic review"<br>meta-analysis<br>cohort<br>cross-over<br>case-control<br>prospective<br>retrospective<br>longitudinal<br>follow-up<br>followup	risk*<br>odds<br>"cross-product ratio*"<br>"hazards ratio*"<br>"hazard ratio*"<br>statistic*<br>"HR"<br>"RR"<br>"aOR"<br>relation*<br>correlat*<br>associat*<br>likel*

For peer review only

	"early marriage*"<br>"child bride*"<br>CEFM<br>"female genital mutilation"<br>"female genital cutting"<br>"female circumcision"<br>"female genital circumcision"<br>infibulation*<br>clitoridectom*<br>clitorectom*<br>"ritual female genital surger*"<br>FGM<br>"physical abuse"<br>"psychological abuse"<br>"emotional abuse"<br>"economic abuse"<br>"financial abuse"<br>"verbal abuse"<br>maltreatment<br>"violent discipline"<br>"corporal punishment"<br>"adverse childhood experience*"<br>molestation<br>"child abuse"<br>"partner abuse"<br>"dating abuse"<br>"wife abuse"<br>"spouse abuse"<br>"domestic abuse"<br>"elder abuse"<br>"senior abuse"<br>"aged abuse"<br>victimization<br>dehumanization<br>victimisation<br>dehumanisation<br>stalking<br>cyberviolence<br>cybervictimization<br>cyberstalking		

For peer review only

Supplementary Table 3. Search Terms for PsycInfo.

	Concept: Violence	Concept: Study type	Concept: Risk
PsycInfo Subject Headings (DE)	DE(<br>"Sexual Violence"<br>"Sex Offenses"<br>"Sexual Abuse"<br>"Sexual Coercion"<br>"Sex Trafficking"<br>"Violence"<br>"Domestic Violence"<br>"Child Abuse"<br>"Elder Abuse"<br>"Circumcision"<br>"Intimate Partner Violence"<br>"Dating Violence"<br>"Physical Abuse"<br>"Physical Discipline"<br>"Punishment"<br>"Rape"<br>"Acquaintance Rape"<br>"Torture"<br>"Workplace Violence"<br>"Gun Violence"<br>"Battered Females"<br>"Exposure to Violence"<br>"Emotional Abuse"<br>"Sexual Harassment"<br>"Aggressive Behavior"<br>"Verbal Abuse"<br>"Coercion"<br>"Stalking"<br>"Childhood Adversity"<br>"School Violence"<br>"Police Violence"<br>"Victimization"<br>)	DE(<br>"Cohort Analysis"<br>"Followup Studies"<br>"Longitudinal Studies"<br>"Retrospective Studies"<br>"Prospective Studies"<br>"Systematic Review"<br>"Meta Analysis"<br>)	DE(<br>"Statistics"<br>"Statistical Analysis"<br>"Risk Assessment"<br>"Statistical Correlation"<br>)
MeSH terms (MA)	MA(<br>"Sex Offenses"<br>"Domestic Violence"<br>"Gender-Based Violence"<br>"Intimate Partner Violence"<br>"Physical Abuse"<br>"Rape"	MA(<br>"Case-Control Studies"<br>"Cross-Over Studies"<br>"Cohort Studies"<br>)	MA(<br>"Statistics as Topic"<br>Risk<br>Odds Ratio<br>)

For peer review only

	"Torture"<br>"Workplace Violence"<br>"Gun violence"<br>"Battered Women"<br>"Adult Survivors of Child abuse"<br>"Exposure to Violence"<br>"Emotional Abuse"<br>"Sexual Harassment"<br>"Coercion"<br>"Dehumanization"<br>"stalking"<br>"adverse childhood experiences"<br>)		
Free text terms (searched in Title, Abstract, and Keywords)	violence<br>"sexual assault"<br>"sexual harassment"<br>"sexual abuse"<br>"sex abuse"<br>rape<br>"forced sex"<br>"sexual coercion"<br>"reproductive coercion"<br>"sex trafficking"<br>"sexual exploitation"<br>"forced marriage*"<br>"child marriage*"<br>"early marriage*"<br>"child bride*"<br>CEFM<br>"female genital mutilation"<br>"female genital cutting"<br>"female circumcision"<br>"female genital circumcision"<br>infibulation*<br>clitoridectom*<br>clitorectom*<br>"ritual female genital surger*"<br>FGM<br>"physical abuse"<br>"psychological abuse"<br>"emotional abuse"<br>"economic abuse"	"systematic review"<br>meta-analysis<br>cohort<br>cross-over<br>case-control<br>prospective<br>retrospective<br>longitudinal<br>follow-up<br>followup	risk*<br>odds<br>"cross-product ratio*"<br>"hazards ratio*"<br>"hazard ratio*"<br>statistic*<br>"HR"<br>"RR"<br>"aOR"<br>relation*<br>correlat*<br>associat*<br>likel*

For peer review only

	"financial abuse"<br>"verbal abuse"<br>maltreatment<br>"violent discipline"<br>"corporal punishment"<br>"adverse childhood experience*"<br>molestation<br>"child abuse"<br>"partner abuse"<br>"dating abuse"<br>"wife abuse"<br>"spouse abuse"<br>"domestic abuse"<br>"elder abuse"<br>"senior abuse"<br>"aged abuse"<br>victimization<br>dehumanization<br>victimisation<br>dehumanisation<br>stalking<br>cyberviolence<br>cybervictimization<br>cyberstalking		

For peer review only

Supplementary Table 4. Search Terms for Global Index Medicus.

	Concept: Violence	Concept: Study type	Concept: Risk
Subject Descriptors	I01.198.240.748*<br>Violence<br>I01.198.240.856.350*<br>I01.198.240.856.463<br>I01.198.240.856.575*<br>I01.198.240.856.688<br>I01.198.240.748.640<br>I01.198.240.856.825<br>I01.198.240.856.912<br>I01.198.240.856.519<br>M01.975.155<br>M01.135.500<br>I01.880.735.900.869<br>I01.880.735.305<br>SP9.020.800.010<br>"Harassment, Non-Sexual"<br>"Aggression"<br>I01.880.604.316<br>Dehumanization<br>F01.145.813.191*<br>I01.880.735.223.500<br>I01.880.735.035	E05.318.372.500.500*<br>E05.318.370.150<br>E05.318.372.500.750*<br>V03.850<br>L01.178.682.759.575<br>V03.600<br>E05.318.370.500*<br>V03.900	E05.318.740*<br>E05.318.740.600.800*<br>E05.318.740.600.600
Title, abstract, subject (tw:())	violence<br>"sexual assault"<br>"sexual harassment"<br>"sexual abuse"<br>"sex abuse"<br>rape<br>"forced sex"<br>"sexual coercion"<br>"reproductive coercion"<br>"sex trafficking"<br>"sexual exploitation"<br>"forced marriage"<br>"forced marriages"<br>"child marriage"<br>"child marriages"<br>"early marriage"<br>"early marriages"<br>"child bride"<br>"child brides"<br>CEFM<br>"female genital mutilation"<br>"female genital cutting"	"systematic review"<br>"meta-analysis"<br>cohort<br>"cross-over"<br>"case-control"<br>Prospective<br>retrospective<br>longitudinal<br>"follow-up"<br>followup	risk*<br>odds<br>"cross-product ratio"<br>"cross-product ratios"<br>"hazards ratio"<br>"hazards ratios"<br>"hazard ratio"<br>"hazard ratios"<br>statistic*<br>"HR"<br>"RR"<br>"aOR"<br>relation*<br>correlat*<br>associat*<br>likel*

	"female circumcision"<br>"female genital circumcision"<br>infibulation*<br>clitoridectom*<br>clitorectom*<br>"ritual female genital surgery"<br>"ritual female genital surgeries"<br>FGM<br>"physical abuse"<br>"psychological abuse"<br>"emotional abuse"<br>"economic abuse"<br>"financial abuse"<br>"verbal abuse"<br>maltreatment<br>"violent discipline"<br>"corporal punishment"<br>"adverse childhood experience"<br>"adverse childhood experiences"<br>molestation<br>"child abuse"<br>"partner abuse"<br>"dating abuse"<br>"wife abuse"<br>"spouse abuse"<br>"domestic abuse"<br>"elder abuse"<br>"senior abuse"<br>"aged abuse"<br>victimization<br>dehumanization<br>victimisation<br>dehumanisation<br>stalking<br>cyberviolence<br>cybervictimization<br>cyberstalking		

For peer review only

Supplementary Table 5. Search Terms for Cochrane.

	Concept: Violence	Concept: Study type	Concept: Risk
Subject Headings [mh].	[mh "Sex Offenses"]. [mh ^"Violence"]. [mh "Domestic Violence"]. [mh "Gender-Based Violence"]. [mh "Intimate Partner Violence"]. [mh "Physical Abuse"]. [mh "Rape"]. [mh "Torture"]. [mh "Workplace Violence"]. [mh "Gun violence"]. [mh "Battered Women"]. [mh "Adult Survivors of Child abuse"]. [mh "Exposure to Violence"]. [mh "Emotional Abuse"]. [mh "Sexual Harassment"]. [mh ^"Harassment, Non-Sexual"]. [mh ^"Aggression"]. [mh "Coercion"]. [mh "Dehumanization"]. [mh "stalking"]. [mh "adverse childhood experiences"].	[mh "Case-Control Studies"]. [mh "Cross-Over Studies"]. [mh "Cohort Studies"]. [mh "Systematic Review"]. [mh "Meta-Analysis"]. [mh "Twin Study"].	[mh "Statistics as Topic"]. [mh Risk]. [mh "Odds Ratio"].
Free text terms :ti,ab,kw	violence "sexual assault" "sexual harassment" "sexual abuse" "sex abuse" rape "forced sex" "sexual coercion" "reproductive coercion" "sex trafficking" "sexual exploitation" ((forced OR child OR early) NEXT marriage*) (child NEXT bride*) CEFM "female genital mutilation" "female genital cutting"	"systematic review" "meta-analysis" "cohort" "cross-over" "case-control" "prospective" "retrospective" "longitudinal" "follow-up" "followup"	"risk" "odds" "cross-product ratio" "cross-product ratios" "hazards ratio" "hazards ratios" "hazard ratio" "hazard ratios" statistic* "HR" "RR" "aOR" relation* correlat* associat* likel*

For peer review only

	"female circumcision"<br>"female genital circumcision"<br>infibulation*<br>clitoridectom*<br>clitorectom*<br>"ritual female genital surgery"<br>"ritual female genital surgeries"<br>FGM<br>((physical OR psychological OR emotional OR economic OR financial OR verbal) NEXT abuse)<br>maltreatment<br>"violent discipline"<br>"corporal punishment"<br>"adverse childhood experience"<br>"adverse childhood experiences"<br>molestation<br>((child OR partner OR dating OR wife OR spouse OR domestic OR elder OR senior OR aged) NEXT abuse)<br>victimization<br>dehumanization<br>victimisation<br>dehumanisation<br>stalking<br>cyberviolence<br>cybervictimization<br>cyberstalking		

For peer review only

Supplementary Table 6. Search Terms for Web of Science.

	Concept: Violence	Concept: Study type	Concept: Risk
Topic (searches title, abstract, author keywords, Keywords Plus®)	violence<br>"sexual assault"<br>"sexual harassment"<br>"sexual abuse"<br>"sex abuse"<br>rape<br>"forced sex"<br>"sexual coercion"<br>"reproductive coercion"<br>"sex trafficking"<br>"sexual exploitation"<br>"forced marriage*"<br>"child marriage*"<br>"early marriage*"<br>"child bride*"<br>CEFM<br>"female genital mutilation"<br>"female genital cutting"<br>"female circumcision"<br>"female genital circumcision"<br>infibulation*<br>clitoridectom*<br>clitorectom*<br>"ritual female genital surger*"<br>FGM<br>"physical abuse"<br>"psychological abuse"<br>"emotional abuse"<br>"economic abuse"<br>"financial abuse"<br>"verbal abuse"<br>Maltreatment<br>torture<br>"violent discipline"<br>"corporal punishment"<br>"adverse childhood experience*"<br>molestation<br>"child abuse"<br>"partner abuse"	"systematic review"<br>"meta-analysis"<br>cohort<br>cross-over<br>case-control<br>prospective<br>retrospective<br>longitudinal<br>follow-up<br>followup	"risk*"<br>"odds"<br>"cross-product ratio*"<br>"hazards ratio*"<br>"hazard ratio*"<br>statistic*<br>"HR"<br>"RR"<br>"aOR"<br>relation*<br>correlat*<br>associat*<br>likel*

For peer review only

	"dating abuse"<br>"wife abuse"<br>"battered wom*n"<br>"spouse abuse"<br>"domestic abuse"<br>"elder abuse"<br>"senior abuse"<br>"aged abuse"<br>victimization<br>dehumanization<br>victimisation<br>dehumanisation<br>stalking<br>cyberviolence<br>cybervictimization<br>cyberstalking		

For peer review only
